# Peptidyl-prolyl cis/trans isomerase Pin1 interacts with hepatitis B virus core particle, but not with HBc protein, to promote HBV replication

**DOI:** 10.3389/fcimb.2023.1195063

**Published:** 2023-06-19

**Authors:** Hyeonjoong Kwon, Jumi Kim, Chanho Song, Muhammad Azhar Sajjad, Jiseon Ha, Jaesung Jung, Sun Park, Ho-Joon Shin, Kyongmin Kim

**Affiliations:** ^1^ Department of Microbiology, Ajou University School of Medicine, Suwon, Republic of Korea; ^2^ Department of Biomedical Science, Graduate School of Ajou University, Suwon, Republic of Korea

**Keywords:** hepatitis B virus, HBV replication, core particle, PPIase Pin1, Pin1-core particle interaction

## Abstract

Here, we demonstrate that the peptidyl-prolyl cis/trans isomerase Pin1 interacts noncovalently with the hepatitis B virus (HBV) core particle through phosphorylated serine/threonine-proline (pS/TP) motifs in the carboxyl-terminal domain (CTD) but not with particle-defective, dimer-positive mutants of HBc. This suggests that neither dimers nor monomers of HBc are Pin1-binding partners. The ^162^TP, ^164^SP, and ^172^SP motifs within the HBc CTD are important for the Pin1/core particle interaction. Although Pin1 dissociated from core particle upon heat treatment, it was detected as an opened-up core particle, demonstrating that Pin1 binds both to the outside and the inside of the core particle. Although the amino-terminal domain S/TP motifs of HBc are not involved in the interaction, ^49^SP contributes to core particle stability, and ^128^TP might be involved in core particle assembly, as shown by the decreased core particle level of S49A mutant through repeated freeze and thaw and low-level assembly of the T128A mutant, respectively. Overexpression of Pin1 increased core particle stability through their interactions, HBV DNA synthesis, and virion secretion without concomitant increases in HBV RNA levels, indicating that Pin1 may be involved in core particle assembly and maturation, thereby promoting the later stages of the HBV life cycle. By contrast, parvulin inhibitors and *PIN1* knockdown reduced HBV replication. Since more Pin1 proteins bound to immature core particles than to mature core particles, the interaction appears to depend on the stage of virus replication. Taken together, the data suggest that physical association between Pin1 and phosphorylated core particles may induce structural alterations through isomerization by Pin1, induce dephosphorylation by unidentified host phosphatases, and promote completion of virus life cycle.

## Introduction

Hepatitis B virus (HBV), a prototype virus belonging to the *Hepadnaviridae* family, is a small, enveloped DNA virus that replicates preferentially in the liver and utilizes a unique replication strategy involving reverse transcription of pregenomic RNA (pgRNA) (Tsukuda and Watashi, 2020). HBV causes acute and chronic hepatitis B; the latter may progress to fibrosis, cirrhosis, or hepatocellular carcinoma (HCC) (Seeger and Mason, 2015). It is estimated that 296 million people are living with hepatitis B virus infection, with 820,000 deaths worldwide (as of 2019, WHO). Although a highly effective vaccine is available, HBV infection remains as a major global health concern. Therefore, to develop novel anti-HBV therapies, it is important to understand how the HBV life cycle is regulated at the cellular and molecular levels.

HBV infects cells via the sodium taurocholate cotransporting polypeptide (NTCP) functional receptor (Yan et al., 2012). After cell entry, the relaxed circular DNA (RC DNA) within the mature core particle (capsid or nucleocapsid) is transported to the nucleus and converted to covalently closed circular DNA (cccDNA). The cccDNA (an episomal DNA) serves as a template for viral transcription. Four major RNA species (pgRNA, the 2.4 and 2.1 kb of S mRNAs, and X mRNA) are transcribed from the cccDNA, transported to the cytoplasm, and translated to produce viral proteins: core (HBc, C, capsid), polymerase (Pol, P), surface (HBs, S), and X (HBx). HBs includes large HBs (LHBs), middle HBs (MHBs), and small HBs (SHBs) proteins. pgRNA is selectively encapsidated into the core particle to serve as a template for reverse transcription to generate viral DNA.

**Figure 1:**
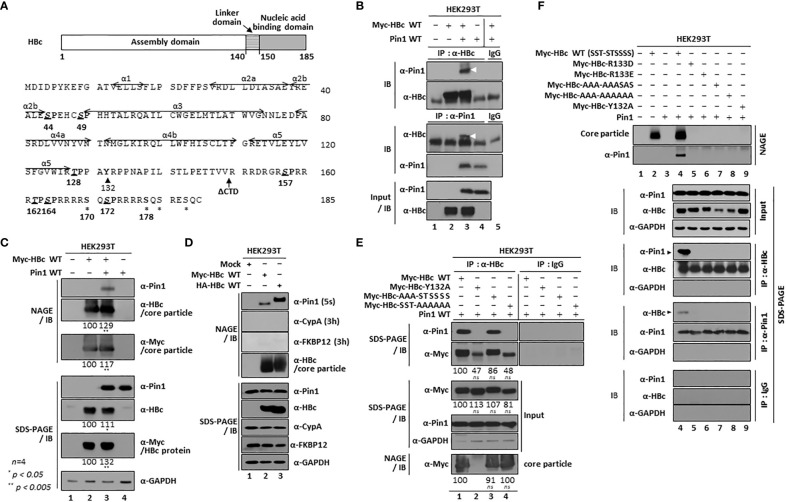
HBV Core Particles, But Not HBc Dimers or Monomers, Interact with Pin1. **(A)** Schematic diagram and amino acid sequence of the HBV HBc (adw). The positions of the helices (Wynne et al., 1999) are shown by arrows. The CTD-truncated (at amino acid 149) HBc used for this study is denoted as ΔCTD. The S/TP motifs are in bold, italicized, and underlined. Y132 and other phosphoacceptor sites are marked by arrowheads and asterisks, respectively. **(B)** Myc-HBc WT and Pin1 WT can be coimmunoprecipitated. HEK293T cells were (co)transfected with mock (lane 1), Myc-HBc WT (lane 2), Myc-HBc WT plus Pin1 WT (lane 3), or Pin1 WT (lane 4). Two days after transfection, cell lysates were immunoprecipitated with an in-house rabbit polyclonal anti-HBc antibody (Jung et al., 2012) and then immunoblotted with a mouse monoclonal anti-Pin1 antibody, or vice versa. As a negative control, normal rabbit IgG or normal mouse IgG was used to examine lysates from Myc-HBc WT plus Pin1 WT cotransfected cells (lane 5). Immunoprecipitated proteins were subjected to SDS-PAGE, followed by immunoblotting with anti-HBc and anti-Pin1 antibodies. Lysates were also subjected to SDS-PAGE, followed by immunoblotting with anti-HBc and anti-Pin1 antibodies. Coimmunoprecipitated proteins are marked with a white arrowhead. **(C)** Pin1 interacts with the core particle. HEK293T cells were (co)transfected as above. Two days after transfection, cell lysates were prepared and subjected to NAGE; transferred to PVDF membranes; and immunoblotted with anti-Pin1, anti-HBc, or mouse monoclonal anti-Myc antibodies. Lysates were also subjected to SDS-PAGE, followed by immunoblotting with anti-HBc, anti-Myc, anti-Pin1, or mouse monoclonal anti-GAPDH antibodies, as described in B. GAPDH was used as a loading control. **(D)** The interaction between Pin1 and the core particle is more specific than that between the core particle and other PPIases. HEK293T cells were transfected with mock (lane 1), Myc-HBc WT (lane 2), or HA-HBc WT (lane 3), and cell lysates were subjected to NAGE plus immunoblotting and SDS-PAGE plus immunoblotting, as described in **(B, C)**. Additionally, blots were probed with rabbit monoclonal anti-CypA and mouse monoclonal anti-FKBP12 antibodies. **(E)** The core particle, not a dimer or monomer of HBc, binds to Pin1 through the CTD phosphoacceptor S/TP motifs of HBc. HEK293T cells were cotransfected with Pin1 WT plus Myc-HBc WT (SST-STSSSS) (lane 1), or with the Myc-HBc-Y132A (lane 2), Myc-HBc-AAA-STSSSS (lane 3), or Myc-HBc-SST-AAAAAA (lane 4) mutants. Lysates were immunoprecipitated and then subjected to SDS-PAGE and immunoblotting as described in B. Core particle immunoblotting after NAGE was also performed as described in **(C)**. **(F)** Pin1 does not interact with core particle-defective, dimer-positive HBc mutants. HEK293T cells were (co)transfected with mock (lane 1), Myc-HBc WT (lane 2), Pin1 WT (lane 3), Pin1 WT plus Myc-HBc WT (lane 4), Myc-HBc-R133D (lane 5), Myc-HBc-R133E (lane 6), Myc-HBc-AAA-AAASAS (lane 7), Myc-HBc-AAA-AAAAAA (lane 8), or Myc-HBc-Y132A (lane 9). Lysates were immunoprecipitated and then subjected to SDS-PAGE and immunoblotting as described in **(B)**. Representative data are shown. Relative levels of core particles and HBc proteins were calculated using ImageJ 1.50b software. Statistical significance was evaluated using Student’s *t* test. *ns*, not significant; ^*^ *P* < 0.05; ^**^ *P* < 0.005, relative to the corresponding control.

The HBV HBc protein comprises of 183 (the ayw subtype) or 185 (the adw subtype) amino acids (**Figure 1A**). Although 140 amino acids of the amino-terminal domain (NTD) are sufficient for core particle assembly (Gallina et al., 1989; Wynne et al., 1999), the linker domain (141–149 amino acids) and the 34 (ayw) or 36 (adw) amino acids comprising of the arginine-rich protamine-like nucleic acid-binding carboxyl-terminal domain (CTD) are important for HBV replication (Köck et al., 2004; Le Pogam et al., 2005; Lewellyn and Loeb, 2011a; Jung et al., 2012; Jung et al., 2014; Liu et al., 2018). Also, the CTD of HBc contains eight putative phosphorylation sites: seven serines and one threonine (**Figure 1A**) (Machida et al., 1991; Yeh and Ou, 1991; Liao and Ou, 1995; Lan et al., 1999; Daub et al., 2002; Köck et al., 2004; Le Pogam et al., 2005; Lewellyn and Loeb, 2011b; Jung et al., 2014). Among these, the conserved phosphoacceptors ^157^Ser, ^164^Ser, and ^172^Ser of the adw subtype (^155^Ser, ^162^Ser, and ^170^Ser of the ayw subtype) are important for core particle formation, RNA packaging (Kann and Gerlich, 1994; Lan et al., 1999; Gazina et al., 2000; Daub et al., 2002), DNA synthesis (Lan et al., 1999; Köck et al., 2004; Melegari et al., 2005; Perlman et al., 2005; Lewellyn and Loeb, 2011b), and subcellular localization (Kann et al., 1999; Wittkop et al., 2010). Additional phosphoacceptors ^162^Thr, ^170^Ser, and ^178^Ser (^160^Thr, ^168^Ser, and ^176^Ser of the ayw subtype) in the HBc protein also contribute to the HBV life cycle at multiple stages, including core particle formation, RNA packaging, and minus and/or plus DNA synthesis, possibly through phosphorylation and dephosphorylation (Jung et al., 2014).

The proline-directed serine/threonine kinase CDK2, or a CDK2-like kinase, can phosphorylate the hepadnaviral HBc protein at serine-proline (SP) sites (^157^Ser-Pro, ^164^Ser-Pro, and ^172^Ser-Pro) (Ludgate et al., 2012). Residue ^162^Thr within the conserved TP motif may also be phosphorylated by CDK2 or a CDK2-like kinase (Jung et al., 2014).

Peptidyl-prolyl cis/trans isomerases (PPIases) regulate protein folding and functions by twisting the backbone of the target protein through cis/trans isomerization (Göthel and Marahiel, 1999; Lu and Zhou, 2007). All organisms, from prokaryotes to mammals, encode PPIase superfamily proteins (Pemberton and Kay, 2005; Unal and Steinert, 2014), which are classified into four families: cyclophilins, FK506-binding proteins (FKBPs), parvulins, and protein Ser/Thr phosphatase 2A (PP2A) activators (PTPA) (Lu and Zhou, 2007).

The human genome contains two parvulin genes: protein interacting with never in mitosis A (NIMA)-1 (*PIN1*) and *PIN4* (Lu et al., 1996; Rulten et al., 1999; Mueller et al., 2006). *PIN1* encodes the Pin1 protein, whereas the *PIN4* gene encodes parvulin14 (Par14) and parvulin17 (Par17) (Lu et al., 1996; Rulten et al., 1999; Mueller et al., 2006). Previously, we reported that Par14 and Par17 interact physically with HBV oncoprotein HBx through conserved RP motifs on HBx to increase the stability of HBx, as thereby HBV replication, in an HBx-dependent manner (Saeed et al., 2019). We also reported that Par14 and Par17 interact with both the core particle and the HBc protein through a conserved RP motif in HBc (Saeed et al., 2021). Through these interactions, the core particle and HBc are stabilized, and HBV replication is increased (Saeed et al., 2021).

Pin1 is a conserved enzyme that functions as a key regulator of cell cycle progression, cell proliferation, transcriptional regulation, and tumorigenesis by binding to many substrates (Liou et al., 2011; Lu and Hunter, 2014; Zhou and Lu, 2016). Pin1 comprises of 163 amino acids and contains unique domains: a WW binding domain, a flexible linker, and isomerase domains (Lu and Zhou, 2007). The N-terminal WW binding domain is responsible for binding to specific substrate proteins that are phosphorylated at S/TP motifs, and the C-terminal isomerase domain functions to catalyze cis/trans isomerization of the bound peptide bond, thereby inducing conformational changes in the binding partners (Liou et al., 2011; Lu and Hunter, 2014; Zhou and Lu, 2016). By doing so, Pin1 regulates protein stability, transcription, transactivation, catalytic activity, phosphorylation status, protein-protein interactions, and/or subcellular localization (Liou et al., 2011; Lu and Hunter, 2014; Zhou and Lu, 2016). Pin1 is frequently overexpressed in many human cancers, including HCC (Pang et al., 2004; Pang et al., 2007; Cheng et al., 2013; Lu and Hunter, 2014; Kim et al., 2015; Shinoda et al., 2015), thereby contributing to centrosome amplification, chromosome instability, and tumorigenesis both *in vitro* and *in vivo*; indeed, its expression correlates with poor clinical outcomes (Zhou and Lu, 2016). HBx binds to Pin1 through the ^41^SP motif on HBx, thereby promoting hepatocarcinogenesis (Pang et al., 2007). A recent study reported that Pin1 and HBc interact through phosphorylated S/TP motifs on HBc, especially p^160^TP and p^162^SP, to increase HBc stability and promote efficient HBV replication (Nishi et al., 2020). In addition to HBx and HBc, other viral proteins bind Pin1 to increase viral replication and/or oncogenesis (Kojima and Ryo, 2010; Hou et al., 2015; Kanna et al., 2022).

However, we show here that Pin1 interacts exclusively with the HBV core particle, possibly through pS/TP motifs on HBc, which is a mechanism different from that reported previously (Nishi et al., 2020). Unlike Par14 and Par17, which bind to both HBc and the core particle (Saeed et al., 2021), Pin1 binds neither dimers nor monomers of HBc. Like Par14 and Par17 (Saeed et al., 2021), we found that Pin1 binds both outside and inside the core particle. Furthermore, we show that the^162^TP, ^164^SP, and ^172^SP motifs on HBc are important for the Pin1/core particle interaction. Since the ^157^SP, ^162^TP, ^164^SP, and ^172^SP motifs are phosphorylated *in vivo* (Kann and Gerlich, 1994; Kau and Ting, 1998; Daub et al., 2002; Chen et al., 2011; Ludgate et al., 2012; Jung et al., 2014) and are important for HBV propagation (Kann and Gerlich, 1994; Lan et al., 1999; Gazina et al., 2000; Köck et al., 2004; Le Pogam et al., 2005; Melegari et al., 2005; Lewellyn and Loeb, 2011b; Jung et al., 2014), these pS/TP motifs may function in the HBV life cycle through the Pin1/core particle interaction. We found that Pin1 overexpression or knockdown (KD) modulated HBV propagation, leading to increased and decreased HBV replication, respectively, indicating that Pin1 is a positive regulator of HBV propagation. Taken together, our results provide evidence that Pin1 plays an important role in the HBV life cycle by interacting with the HBV core particle, suggesting that not only the Pin1/HBx interaction (Pang et al., 2007) but also the Pin1/core particle interaction may be involved in HBV-associated hepatocarcinogenesis.

## Materials and methods

### Plasmids

To generate an HBV replication competent plasmid, a 1.3-fold over length fragment of HBV wild-type (WT) subtype adw R9 genome was cloned into the pGEM4z (Promega) vector and then subcloned to pcDNA3; the resulting construct was designated HBV WT 1.3mer (adw). HBV WT, the priming reaction-deficient TP-Y65F mutant, the RT reaction-deficient RT-YMHA mutant, and the P-deficient mutant, in which transcription of pgRNA is controlled by the cytomegalovirus immediate early (CMV IE) promoter (Kim et al., 2004), were used for the study. Also, an HBx-deficient mutant, in which HBx expression was abolished completely by mutation of three ATGs in the HBx open reading frame, was placed under the control of the CMV IE promoter (Yoon et al., 2011). A subtype ayw 1.3mer HBV WT plasmid was kindly provided by Dr. Ryu WS (Yonsei Uni, South Korea). With the exception of the ayw 1.3mer HBV WT plasmid, all of the HBV constructs used in this study had the adw HBV background. Myc- or HA-tagged HBc WT was constructed by PCR-amplification of an HBc sequence on a P-deficient mutant background (Kim et al., 2004; Jung et al., 2014); the primers used are listed in the Table. The amplified PCR product was digested with *Msc* I and *Kpn* I, gel-purified, and inserted into a pCMV10-Myc or pCMV-HA plasmid to generate pCMV10-Myc-HBc WT (SST-STSSSS) or pCMV10-HA-HBc WT, respectively. Myc-tagged HBc mutants were constructed by PCR-generated site directed mutagenesis of pCMV10-Myc-HBc WT, thereby generating pCMV10-Myc-HBc-Y132A, pCMV10-Myc-HBc-SST-SAAAAA, pCMV10-Myc-HBc-SST-AASAAA, pCMV10-Myc-HBc-SST-AAAASA, pCMV10-Myc-HBc-S157A, pCMV10-Myc-HBc-T162A, pCMV10-Myc-HBc-S164A, pCMV10-Myc-HBc-S172A, pCMV10-Myc-HBc-S44A, pCMV10-Myc-HBc-S49A, pCMV10-Myc-HBc-T128A, and pCMV10-Myc-HBc-AAA-STSSSS. The sequences of the primers used to construct the HBc WT and HBc mutants are listed in the Table. To generate the Myc-tagged HBc-SST-ATAAAA and HBc-SST-AAAAAA mutants, mutant HBc DNA fragments from previously published ATAAAA and AAAAAA mutants of HBc on a P-deficient mutant background (Jung et al., 2014) were inserted into a *Msc* I/*Kpn* I-linearized pCMV10-Myc plasmid to yield pCMV10-Myc-HBc-SST-ATAAAA and pCMV10-Myc-HBc-SST-AAAAAA, respectively. To generate the Myc-tagged HBc-AAA-AAAAA and -AAA-AAASAS mutants, site directed mutagenesis was conducted using the primers listed in the Table, and the mutant HBc DNA PCR fragment was inserted into a *Msc* I/*Kpn* I-linearized pCMV10-Myc plasmid to yield pCMV10-Myc-HBc-AAA-AAAAA and pCMV10-Myc-HBc-AAA-AAASAS, respectively. HBc-ΔCTD comprising the NTD 145 amino acids of HBc has been described (Jung et al., 2012). To generate a WT Pin1 expression construct, the *PIN1* cDNA was synthesized from total RNA of HepG2 cells using superscript III reverse transcriptase (18080-044, Invitrogen™) and a reverse primer specific for *PIN1* WT. Next, the cDNA was amplified by PCR using the forward and reverse primers for *PIN1* WT (Table). The amplified DNA fragment was inserted into *Hin*d III/*Bam*H I linearized pcDNA3.1 (Invitrogen™). PCR-generated site directed mutagenesis was used to construct dephosphorylation mimetic substrate binding-positive S16A (AGC → GCC), phosphorylation mimetic substrate binding-deficient S16E (AGC → GAA), substrate binding-deficient W34A (TGG → GCT), PPIase-active S71A (TCG → GCG), and PPIase-inactive C113A (TGC → GCC) mutants (Table). The resulting Pin1 mutant plasmids were designated as pcDNA3-Pin1-S16A, pcDNA3-Pin1-S16E, pcDNA3-Pin1-W34A, pcDNA3-Pin1-S71A, and pcDNA3-Pin1-C113A. The pCDH-CMV-*PIN1*-EF1-Puro plasmid encoding *PIN1* in a lentiviral vector was generated by inserting the amplified *PIN1* WT DNA fragment into the *Eco*R I and *Bam*H I sites of pCDH-CMV-MCS-EF1-Puro (System Biosciences). Four short hairpin (sh)RNA (shPIN1 #1, 2, 3, and 4) constructs in the pLK0.1 vector, which target multiple regions of the *PIN1* gene, were purchased from Sigma-Aldrich (SHCLNG-NM_006221). Control shRNA harboring random sequences (5’-ACG TGA CAC GTT CGG AGA AC) was inserted into pLK0.1 to generate a scrambled shRNA control. The primer sequences used to construct *PIN1* WT and its mutants, and the shRNAs targeting *PIN1* are listed in the Table. All constructs were sequenced to confirm the presence of the introduced mutations and the absence of extraneous mutations.

### Cell culture and transfection

HepG2, Huh7, or HEK293T cells were grown in Dulbecco’s modified Eagle’s medium (DMEM) supplemented with 10% fetal bovine serum (FBS; Gibco-BRL) and 1% penicillin/streptomycin under a humidified atmosphere at 37°C/5% CO_2_. HepG2 and Huh7 cells and HEK293T cells were passaged every third and second day, respectively. HepG2.2.15 cells were grown in the same culture medium as HepG2 cells, except for addition of 0.2 mg/ml G418 (G0175.0005, Duchefa Biochemie). HepAD38 cells (kindly provided by Christopher Seeger, Fox Chase Cancer Center) were also grown in the same culture medium as HepG2.2.15 cells, except that tetracycline (1 µg/ml, T7660-5G, Sigma-Aldrich) was added. The tetracycline was removed to induce HBV transcription and production of HBV virions. For transfection into 1×10^6^ HEK293T cells in 6 cm culture plates, 2 µg of each construct was mixed with 4 µg/µl polyethylenimine (PEI; Polysciences) in 200 µl of OPTI-MEM (Gibco). For transfection into 2×10^6^ HepG2 or HepG2.2.15 cells in 6 cm culture plates, 3 µg of each construct was mixed with 9 µg/µl PEI in 200 µl of OPTI-MEM and poured onto the cells. For transfection into 1×10^6^ Huh7-Pin1 cells in 6 cm culture plates, 3 µg of each construct was mixed with 9 µg/µl PEI in 200 µl of OPTI-MEM and poured onto the cells. For cotransfection into HEK293T cells in 6 cm culture plates, 2 µg of Pin1-expressing plasmid plus 2 µg of HBV WT, HBc WT, HBc-ΔCTD, Myc-tagged HBc WT, Myc-tagged HBc S/TP site mutants, Myc-tagged-HBc-R133D mutant, Myc-tagged-HBc-R133E mutant, or Myc-tagged HBc-Y132A mutant were mixed with 8 µg/µl PEI in 200 µl of OPTI-MEM and poured onto the cells. For cotransfection into HepG2 cells in 6 cm culture plates, 3 µg of 1.3mer HBV WT plus 3 µg of Pin1 WT or empty pcDNA3.1 vector was mixed with 18 µg/µl PEI in 200 µl of OPTI-MEM and poured onto the cells. Also, HepG2 cells were cotransfected with a mixture of 3 µg of Pin1 WT plus 3 µg of HBV WT (adw) or HBx-deficient HBV (adw) mutant, plus 18 µg/µl PEI in 200 µl of OPTI-MEM. The amount of pcDNA3 was adjusted to the amount of transfected DNA. At 24 h post-transfection, the culture medium containing transfected DNA was exchanged for fresh complete medium.

### Establishment of stable cell lines

Huh7 and HepAD38 cells stably expressing human Pin1 were generated using a lentiviral expression system. Briefly, 2×10^6^ HEK293T cells were seeded on 10 cm plates and transfected with 1 µg of pVSV-G, 3 µg of pGag-Pol, and 4 µg of pCDH-CMV-Pin1-EF1-Puro in 16 µg of PEI/500 µl of OPTI-MEM. One day after transfection, the medium was replaced, and 2 days after transfection, the supernatant containing the pseudoviral particles containing Pin1-transcripts was harvested. Next, 1 ml of supernatant containing pseudoviral particles was mixed with 3 ml of fresh complete medium containing 10 µg/ml polybrene (Hexadimethrine bromide, Sigma-Aldrich) and inoculated onto Huh7 or HepAD38 cells in 6 cm plates. After transduction with lentiviral pseudoviral particles, human Pin1 WT-expressing Huh7 or HepAD38 cells were established by selection with 10 µg/ml puromycin (Sigma-Aldrich) for 72 h. To generate *PIN1* KD HepG2 and *PIN4* KD HEK293T cells, HEK293T cells were transfected with 1 µg of pVSV-G, 3 µg of pGAG-Pol, and 4 µg of pLK0.1-shPIN1 (#1, 2, 3, and 4); pLK0.1-shPIN4 #1; or pLK0.1-shControl in 16 µg of PEI/500 µl of OPTI-MEM. Then, HepG2 or HEK293T cells were transduced with supernatant containing pseudoviral particles and shRNAs (shPIN1 or shPIN4) or control shRNA, as mentioned above, and then selected with puromycin. Stable HepG2-hNTCP-C9 cells were generated as described previously using lentiviral expression vector pCDH-CMV-hNTCP-C9-EF1-Puro (Kim et al., 2022). HepG2-hNTCP-C9-derived *PIN1* knockdown cells were generated as described above.

### Immunoprecipitation, SDS-PAGE, and immunoblotting

For immunoprecipitation, HEK293T cells were (co)transfected with Myc-HBc WT, Pin1 WT, or Myc-HBc WT plus Pin1 WT. Also, HEK293T cells were cotransfected with Pin1 WT plus the Myc-HBc WT (SST-STSSSS) or Myc-HBc mutants (Y132A, AAA-STSSSS, SST-AAAAAA). At 2 days post-transfection, cells were harvested, lysed in 0.2% NP-40 (IGEPAL, Sigma-Aldrich)-TNE buffer (10 mM Tris-HCl [pH 8.0], 50 mM NaCl, and 1 mM EDTA) containing 1 mM PMSF (P7626, Sigma-Aldrich), as previously described (Kim et al., 2004); immunoprecipitated with a rabbit polyclonal anti-HBc (produced in-house; 1:1,000) (Jung et al., 2012); and then immunoblotted with mouse monoclonal anti-Pin1 (1:1,000; sc-46660; Santa Cruz) antibodies, or vice versa. Rabbit or mouse normal IgG was used as a negative control for immunoprecipitation (12-370, 12-371, Merck-Millipore). To analyze the structural stability of the HBc NTD S/TP motif mutants, the cell lysates were exposed to freezing temperatures (approximately -20°C) for 16 h, followed by thawing at room temperature for 1 h. This freeze-thaw cycle was repeated two or four times. For reducing SDS-PAGE, sample buffer containing 125 mM Tris-HCl [pH 6.8], 20% glycerol, 4% SDS, 0.1% bromophenol blue, and 5% β-mercaptoethanol was used. For nonreducing PAGE, the sample buffer was as described above, except that the 5% β-mercaptoethanol was omitted (Saeed et al., 2021). For SDS-PAGE and immunoblotting, equal quantities of protein from cell lysates were subjected to SDS-PAGE on 13.5% gels, transferred to polyvinylidene fluoride (PVDF) membranes (Millipore), and then incubated with the appropriate primary antibodies: rabbit polyclonal anti-HBc (produced in-house; 1:1,000) (Jung et al., 2012), mouse monoclonal anti-Myc (1:1,000; sc-40; Santa Cruz), mouse monoclonal anti-Pin1 (1:1,000; Santa Cruz), rabbit monoclonal anti-CypA (1:1,000; ab126738; Abcam), mouse monoclonal anti-FKBP12 (1:1,000; sc-133067; Santa Cruz), mouse monoclonal anti-GAPDH (1:1,000; sc-32233; Santa Cruz), rabbit monoclonal anti-PIN4 (1:1,000; ab155283; Abcam), rabbit polyclonal anti-HBs (1:1,000; 1811; Virostat) for NAGE, mouse monoclonal anti-HBs (1:1,000; sc-53299; Santa Cruz) for SDS-PAGE, mouse monoclonal anti-PreS1 (1:1,000; sc-57761; Santa Cruz), or mouse monoclonal anti-rhodopsin C9 (1:1,000; MAB5356; Millipore). Primary antibodies were followed by anti-rabbit (HRP; 1:5,000 dilution; #31460; Thermo Fisher Scientific) or anti-mouse (HRP; 1:5,000 dilution; 5220-0460; Seracare) secondary antibodies coupled to horseradish peroxidase. The blots were then visualized by enhanced chemiluminescence (ECL™ western blotting detection reagent, Amersham™). Relative band intensities were measured using ImageJ. 1.50b.

### Native agarose gel electrophoresis and immunoblotting

To analyze HBV core particles by immunoblotting, cell lysates in 0.2% NP-40-TNE buffer were electrophoresed on 1% native agarose gels, and the resolved core particles were transferred to PVDF membrane for immunoblotting with a rabbit polyclonal anti-HBc primary antibody (produced in-house; 1:1,000) (Jung et al., 2012), followed by an anti-rabbit secondary antibody conjugated to HRP (1:5,000; Thermo Fisher Scientific). The bound secondary antibodies were visualized by ECL. Relative band intensities were measured using ImageJ. 1.50b.

### Analysis of extracellular HBV particles

HBV particles were prepared from culture supernatants of HepAD38-pCDH, HepAD38-Pin1, and HBV-infected HepG2-hNTCP-C9 cells as described previously (Watashi et al., 2013; Ko et al., 2014; Kim et al., 2022), with minor modifications. Culture supernatants comprising F-12 DMEM (11320-033, Gibco) supplemented with 10% FBS, 1% penicillin/streptomycin, 5 μg/ml insulin (#I9278, Sigma-Aldrich), and 50 μM hydrocortisone hemisuccinate (#1319002, Sigma-Aldrich) were collected 4 days after tetracycline removal, centrifuged briefly, and filtered through a 0.45 μm bottle top filter to remove cell debris. The filtered supernatants were layered onto a 20% sucrose cushion (w/w in TNE buffer) and then ultracentrifuged (Optima L-90K, Beckman Coulter) at 26,000 rpm (115,633 *g*) for 2 h at 4°C. Pellets containing virions, subviral particles, and naked core particles were resuspended in TNE buffer, electrophoresed on 1% native agarose gels, transferred to a PVDF membrane, and immunoblotted. Core particles, virions, Pin1-bound-core particles, and/or subviral particles were detected using rabbit polyclonal anti-HBc (produced in-house; 1:1,000) (Jung et al., 2012), mouse monoclonal anti-PreS1 (1:1,000; Santa Cruz), rabbit polyclonal anti-HBs (for NAGE; 1:1,000; Virostat), mouse monoclonal anti-HBs (for SDS-PAGE; 1:1,000; Santa Cruz), or mouse monoclonal anti-Pin1 (1:1,000; Santa Cruz) primary antibodies, followed by HRP–conjugated anti-rabbit (1:5,000; Thermo Fisher Scientific) or anti-mouse (1:5,000; Seracare) secondary antibodies. The bound secondary antibodies were visualized by ECL. The relative band intensities were measured using ImageJ. 1.50b.

### HBV infection

The HBV inoculum used for infection was harvested from the culture supernatant of HepAD38 cells as described previously, with minor modifications (Watashi et al., 2013; Ko et al., 2014; Kim et al., 2022). Briefly, the culture supernatants were collected every 2-3 days, centrifuged, and filtered as described above. For partial purification of HBV particles, the filtered supernatants were ultracentrifuged through a 20–60% discontinuous sucrose gradient at 26,000 rpm for 3 h at 4°C. Next, the HBV fraction at the interface (between 30–50%) was collected and then pelleted through a 20% sucrose cushion by ultracentrifugation at 26,000 rpm for 1.5 h at 4°C. The amount of HBV DNA in the precipitate after ultracentrifugation was quantitated by southern blotting. For HBV infection, 2×10^5^ HepG2-hNTCP-C9 cells were seeded onto collagen-coated (#354249, Corning) 6-well plates and infected with 2×10^3^ genome equivalents (GEq) of HBV per cell in medium containing 4% polyethylene glycol (#25322-68-3, Affymetrix) and 2.5% dimethyl sulfoxide (DMSO), as described previously (Ni et al., 2014). One day after infection, the cells were washed thoroughly with 1× PBS (at least three times) to remove residual HBV and then maintained in the same fresh medium containing 2.5% DMSO, as described previously (Ni et al., 2014). The medium was changed every second day. Lysates were prepared at 9 days postinfection and subjected to SDS-PAGE and immunoblotting, native agarose gel electrophoresis (NAGE) and immunoblotting, and southern blot analyses. For northern blot analysis, total cell lysates were prepared at 5 days postinfection.

### Interaction between the core particle and Pin1

To study the interaction between Pin1 and the core particle, HEK293T cells were cotransfected with Myc-HBc plus empty vector or with Pin1 WT. At 48 h post-transfection, the lysates were either left untreated (control) or heated at 65°C for 2 h to dissociate bound Pin1 from the core particle, followed by incubation for 5 min on ice. Then, lysates were subjected to NAGE plus immunoblotting, and SDS-PAGE plus immunoblotting, as described above. To detect Pin1 inside the core particle, the core particle on the PVDF membrane was treated with 0.2 N NaOH for 1 min to open it up, followed by UV-crosslinking (XL-1500 UV CROSSLINKER, Spectrolinker™) and immunoblotting with anti-Pin1, anti-HBc, and anti-Myc antibodies.

### Pin1/Core particle interactions and phosphorylation dependency *in vitro*

HBV WT-transfected HEK293T cell lysates containing core particles were prepared as described above and treated for 1 h at 37°C with 1 unit of calf intestinal alkaline phosphatase (CIAP) (#290S, NEB) to remove phosphates from the core particles. Mock-treated HBV WT-transfected HEK293T cell lysates incubated for 1 h at 37°C in the absence of CIAP were used as a positive control. The reactions were stopped by heating to 75°C for 10 min. Pin1-overexpressing lysates were mixed with either mock-treated lysates or CIAP-treated lysates and then further incubated for 0, 12, 24, and 48 h at 37°C, followed by NAGE plus immunoblotting and SDS-PAGE plus immunoblotting, as described above.

### Stability of the core particle

*PIN4*-KD HEK293T cells (5×10^5^) were plated onto 6-well plates and transfected with HBV WT (Kim et al., 2004) plus the mock, Pin1 WT, or Pin1 W34A mutant. At 12 h post-transfection, 50 µg/ml cycloheximide (C1988-1G, Sigma-Aldrich) was added to inhibit protein synthesis, and cell lysates were prepared at 0, 12, 24, and 48 h post-treatment for NAGE plus immunoblotting (for core particle analysis) and SDS-PAGE plus immunoblotting (for analysis of HBc and Pin1 proteins).

### Southern, northern, and *in situ* nucleic acid blotting

To analyze HBV DNA synthesis by southern blotting, HBV DNA extracted from isolated core particles was separated by agarose gel electrophoresis, transferred to a nylon membrane (11417240001, Sigma-Aldrich), and hybridized to a ^32^P-labeled random-primed probe specific for the full-length HBV sequence, as described previously (Jung et al., 2014). To analyze HBV RNA expression by northern blotting, total RNA was extracted using TRIzol reagent (15596026, Thermo Fisher Scientific). In brief, 20 μg of total RNA was denatured at 65°C for 10 min and then electrophoresed on 1% agarose gels (16500500, Thermo Fisher Scientific) containing formaldehyde (F8775, Sigma-Aldrich) and 1× morpholinepropanesulfonic acid (MOPS) buffer [200 mM MOPS, 10 mM EDTA, 50 mM sodium acetate (pH 7.0)]. RNA was then transferred to a nylon membrane and hybridized at 68°C for 4 h to a ^32^P-labeled random-primed probe specific for the full-length HBV sequence. *In situ* nucleic acid blotting was performed to analyze HBV DNAs inside virions and/or naked core particles. Briefly, pellets containing virions and naked core particles were resuspended in TNE buffer, and cell lysates containing cytoplasmic core particles in 0.2% NP-40-TNE buffer were electrophoresed on 1% native agarose gels prior to transfer to a PVDF membrane. The PVDF membrane was treated with 1% SDS for 1 h, followed by 0.2 N NaOH for 30 s. It was then washed quickly with distilled water, UV-crosslinked (XL-1500 UV CROSSLINKER, Spectrolinker™), and hybridized with a ^32^P-labeled random-primed probe specific for the full-length HBV sequence. *In situ* nucleic acid blotting was also performed to detect minus-strand DNA in cytoplasmic core particles using a synthesized Dig-labeled plus-strand RNA probe specific for the full-length HBV sequence. Relative band intensities were measured using ImageJ. 1.50b.

### Extraction of cccDNA

To examine the effects of Pin1 on formation of HBV cccDNA, the latter was extracted using a Hirt protein-free DNA extraction procedure, as described previously (Cai et al., 2013) but with minor modifications. In brief, 3×10^6^ HepG2-hNTCP-C9 cells were seeded into collagen-coated 10 cm dishes and infected with 2×10^3^ GEq HBV, as described above. At 7 days postinfection, the cells were lysed for 30 min at room temperature (on a shaker) with 0.6% SDS-TE buffer (10 mM Tris-HCl [pH 7.5], 10 mM EDTA). The lysates were treated with 5 M NaCl to adjust the final NaCl concentration to 1 M, incubated for 16 h at 4°C, and centrifuged at 14,500 *g* for 30 min. The supernatants were extracted twice with phenol, followed by once with phenol-chloroform, and then precipitated with ethanol for 16 h at room temperature. Finally, the precipitates were analyzed by southern blotting. To further verify the authenticity of HBV cccDNA, the Hirt DNA sample was heated to 85°C for 5 min. Among the three types of HBV DNAs [cccDNA (2.1 Kbp), DL DNA (3.2 Kbp), and protein-free RC DNA (more than 3.2 Kbp)] in the Hirt protein-free DNA extracted sample, RC and DL DNA, but not cccDNA, were denatured. The electrophoretic mobility of cccDNA is not altered by heat treatment. The heat-treated DNA samples were then digested with *Eco*R I to linearize the cccDNA and yield genome-length double-stranded DNA (3.2 Kbp).

## Results

### The HBV core particle is a novel binding substrate of Pin1

The four HBc CTD S/TP motifs (^157^SP, ^162^TP, ^164^SP, and ^172^SP) are phosphoacceptor sites (**Figure 1A**) (Kann and Gerlich, 1994; Lan et al., 1999; Gazina et al., 2000; Melegari et al., 2005; Lewellyn and Loeb, 2011b; Ludgate et al., 2012; Jung et al., 2014) that can serve as Pin1-binding sites (Nishi et al., 2020). To verify the physical interaction between Pin and HBc proteins (Nishi et al., 2020), we performed coimmunoprecipitation experiments (**Figure 1B**) using (co)transfected cell lysates. Lysates were immunoprecipitated with an anti-HBc antibody and immunoblotted with an anti-Pin1 antibody, and vice versa (**Figure 1B**). The anti-HBc and anti-Pin1 antibodies coimmunoprecipitated Pin1 and HBc, respectively (**Figure 1B**, lane 3), confirming the interaction between Pin1 and HBV HBc protein (Nishi et al., 2020). However, coimmunoprecipitation, SDS-PAGE, and immunoblotting cannot discriminate Pin1/HBc interactions from Pin1/core particle interactions.

Therefore, to examine whether the HBV core particle is a binding substrate of Pin1, we performed NAGE of cotransfected cell lysates, followed by immunoblotting (**Figure 1C**). We found that Pin1 bound to the core particle (**Figure 1C**, top panel; lane 3). Thus, Pin1 can interact physically with HBV core particles, a finding not reported before. However, we showed previously that parvulin proteins Par14 and Par17 interact with both HBc and the core particle (Saeed et al., 2021). It should be noted that when we overexpressed Pin1, the levels of both HBc and the core particle increased (**Figure 1C**, second, third, fifth, and sixth panels; lane 2 vs. 3).

Since PPIases bind to Xaa-Pro motifs (Hanes, 2015), and HBc contains 16 Xaa-Pro motifs, we performed NAGE and immunoblotting to examine whether the core particle binds to cyclophilins and/or FKBPs. The Pin1/core particle interaction was detected after an exposure time of only 5 sec (**Figure 1D**, top panel); however, the cyclophilin A/core particle interaction could not be detected even after a long exposure (3 h) (**Figure 1D**, second panel), indicating that cyclophilin A cannot bind to the core particle. By contrast, FKBP12/core particle binding was weakly detectable after a long exposure (3 h) (**Figure 1D**, third panel). These results indicate that the interaction between Pin1 and the core particle is more specific than that between the core particle and other PPIases, suggesting that Pin1 may contribute to HBV proliferation via its interaction with the core particle.

Although we demonstrated the physical interaction between Pin1 and the core particle for the first time, we did not differentiate the Pin1/HBc interaction from the Pin1/core particle interaction (**Figures 1B−D**). Therefore, to check whether Pin1 interacts with HBc, we used a core particle assembly-defective, dimer-positive ^132^Tyr to Ala (Y132A) mutant of HBc (Bourne et al., 2009) in a coimmunoprecipitation experiment with an anti-HBc antibody, followed by immunoblotting with anti-Pin1 antibody. Although expression of the Myc-HBc-Y132A mutant protein was comparable with that of Myc-HBc WT (**Figure 1E**, third panel; lane 1 vs. 2), core particle assembly by Myc-HBc-Y132A was defective, whereas that by Myc-HBc WT was intact (**Figure 1E**, bottom panel; lane 1 vs. 2) (Bourne et al., 2009). As shown in **Figure 1B**, Pin1 coimmunoprecipitated with Myc-HBc WT but not with the core particle-defective Myc-HBc-Y132A mutant (**Figure 1E**, top panel; lane 1 vs. 2), demonstrating that neither dimers nor monomers of HBc are binding partners of Pin1. This clearly demonstrates that the HBV core particle is a *bona fide* binding substrate of Pin1.

To further identify the importance of the S/TP motifs in the NTD and CTD of HBc WT (^44^S^49^S^128^T-^157^S^162^T^164^S^170^S^172^S^178^S, shortened to SST-STSSSS), Myc-tagged HBc-AAA-STSSSS- or Myc-tagged HBc-SST-AAAAAA-transfected lysates were immunoprecipitated with the anti-HBc antibody and then immunoblotted with the anti-Pin1 antibody (**Figure 1E**, top panel; lanes 1 vs. 3 and 4). Unlike Myc-tagged HBc WT and Myc-tagged HBc-AAA-STSSSS, Myc-tagged HBc-SST-AAAAAA was not immunoprecipitated, demonstrating that CTD phosphoacceptor S/TP motifs are important for Pin1-binding (**Figure 1E**, top panel; lanes 1 and 3 vs. 4). It should be noted here that the intensity of the Myc-HBc-Y132A and Myc-HBc-SST-AAAAAA bands detected by the anti-HBc and anti-Myc antibodies was weaker than that of the Myc-HBc WT and Myc-HBc-AAA-STSSSS bands (**Figure 1E**, second panel; lanes 1, 3 vs. 2, 4).

Previously, we showed that the HBc ^133^Arg to Asp (HBc-R133D) and HBc ^133^Arg to Glu (HBc-R133E) mutants were particle assembly-defective and dimer-positive (Saeed et al., 2021), as is HBc-Y132A. Consistent with this, NAGE and immunoblotting revealed that HBc-R133D and -R133E are particle-defective mutants (Saeed et al., 2021) (**Figure 1F**, top panel; lane(s) 2 and 4 vs. 5, 6, and 9). Additionally, when we analyzed the HBc-AAA-AAASAS and HBc-AAA-AAAAAA mutants in which seven S/TP motifs were substituted with AP, we found that they were particle-defective (**Figure 1F**, top panel; lane(s) 2 and 4 vs. 5-9), indicating that HBc S/TP motifs may play a role in core particle assembly. Coimmunoprecipitation with an anti-HBc antibody followed by immunoblotting with an anti-Pin1 antibody, and vice versa, did not detect particle-defective HBc proteins (**Figure 1F**, sixth and ninth panels; lanes 4 vs. 5-9), further demonstrating that neither monomers nor dimers of HBc are binding partners of Pin1.

### Dynamic on-off interaction between Pin1 and the HBV core particle

**Figure 2:**
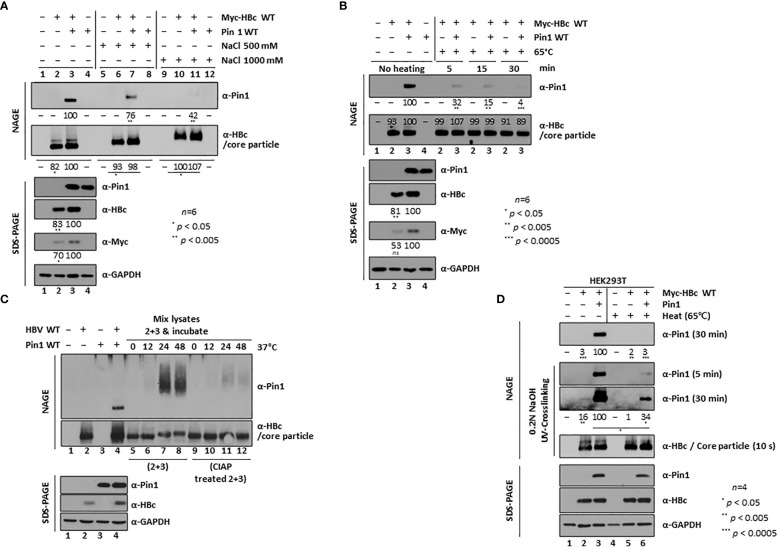
Dynamic On-off Interaction Between Pin1 and the Core Particle. **(A, B)** High salt **(A)** and high temperature **(B)** conditions weaken the Pin1**/**core particle interaction. HEK293T cells were (co)transfected with mock (lane 1), Myc-HBc WT (lane 2), Myc-HBc WT plus Pin1 WT (lane 3), or Pin1 WT (lane 4). Cell lysates prepared at 2 days post-transfection were treated with 500 mM or 1,000 mM NaCl **(A)**, or heated at 65°C for 5, 15, or 30 min **(B)**. **(C)** The *in vitro* interaction between Pin1 and core particle requires phosphorylation of the core particle. HEK293T cells were (co)transfected with mock (lane 1), HBV WT (lane 2), Pin1 WT (lane 3), or HBV WT plus Pin1 WT (lane 4). Pin1 WT-transfected cell lysates and HBV WT-transfected cell lysates were mixed and incubated for the indicated times (C, lanes 5−8), or HBV WT-transfected lysates were treated with CIAP and then mixed with Pin1 WT-transfected lysates for the indicated times (C, lanes 9−12). **(D)** Pin1 binds both outside and inside HBV core particles. HEK293T cells were (co)transfected with mock (lanes 1 and 4), Myc-HBc WT plus 3×FLAG (lanes 2 and 5), or Myc-HBc WT plus Pin1 WT (lanes 3 and 6). At 48 h post-transfection, lysates were either left unheated (lanes 1−3) or heated at 65°C for 2 h (lanes 4−6), followed by NAGE plus immunoblotting and SDS-PAGE plus immunoblotting as described in **Figure 1**. Core particles bound to the PVDF membrane were treated with 0.2N NaOH for 1 min to open them up, crosslinked with UV, and immunoblotted with anti-HBc or anti-Pin1 antibodies. The indicated lysates were subjected to NAGE plus immunoblotting, and to SDS-PAGE plus immunoblotting, as described in **Figure 1**. Representative data are shown. Relative levels of Pin1-bound core particles, core particles, and HBc proteins were calculated using ImageJ 1.50b software. Statistical significance was evaluated using Student’s *t* test. *Ns*, not significant; ^*^ *P* < 0.05; ^**^ *P* < 0.005; ^***^ *P* < 0.0005, relative to the corresponding control.

To characterize Pin1/core particle binding, we treated transfected cell lysates with 0.5 or 1 M sodium chloride and immediately subjected them to NAGE, followed by immunoblotting with anti-Pin1, anti-HBc, and anti-Myc antibodies (**Figure 2A**). As mentioned above, HBc levels in Pin1 WT and Myc-HBc WT cotransfected cells (**Figure 2A**, fourth and fifth panels; lane 2 vs. 3) were slightly higher than Myc-HBc WT transfected cells, and core particle levels increased accordingly (**Figure 2A**, second panel; lane 2 vs. 3). In the presence of high concentrations of sodium chloride, Pin1/core particle binding was significantly weakened (**Figure 2A**, top panel; lanes 3 vs. 7 and 11), indicating that Pin1/core particle binding is a dynamic on-off interaction. Also, core particle levels increased, and the core particle in the presence of high concentrations of sodium chloride migrated more slowly on NAGE than the core particle in untreated lysates (**Figure 2A**, second panel; lanes 2 and 3 vs. 6 and 7 vs. 10 and 11). We think that this is due to increased levels of unstable core particles in the presence of high concentrations of sodium chloride (Jung et al., 2012).

Furthermore, when we incubated transfected lysates at 65°C for 5, 15, and 30 min, the Pin/core particle interaction began to break down at 5 min, and the complex dissociated almost completely after 30 min, further demonstrating that the Pin1/core particle interaction is dynamic (**Figure 2B**, top panel).

Since we used cotransfected lysates to detect the physical Pin1/core particle interaction, we next tried to examine the interaction *in vitro*. To do this, we separately transfected HBV WT and Pin1, and the respective lysates were mixed and incubated for 0, 12, 24, and 48 h at 37°C. Although HBV WT-transfected lysates may contain endogenous Pin1, which might interfere with exogenously added Pin1 in Pin1-transfected lysates, we detected slowly migrating Pin1 by NAGE conducted after 24 and 48 h of incubation; Pin1 detection was independent of transfection, indicating that Pin1 binds to core particles *in vitro* (**Figure 2C**, top panel; lanes 7 and 8). To further show that this *in vitro* interaction depends on the phosphorylation status of the core particle, we treated HBV WT-transfected lysates for 1 h with CIAP. We found that we could hardly detect slowly migrating Pin1 (**Figure 2C**, top panel; lanes 11 and 12), indicating that the physical Pin1/core particle interaction is phosphorylation-dependent. This dynamic on-off and *in vitro* interaction between the core particle and Pin1 (**Figures 2A-C**) indicates that Pin1 binds to the outside of the core particle. This result suggests that some CTDs within HBc, if not all, may face outward from the center of the core particle and serve as binding sites for Pin1.

### Pin1 binds both to the outside and inside of the HBV core particle

Although Pin1 can bind to the outside of the core particle through a dynamic on-off interaction (**Figures 2A-C**), we could not exclude the possibility that Pin1 may also be incorporated into the core particle, like Par14 and Par17 (Saeed et al., 2021). To test this, lysates were mock-treated or heated at 65°C for 2 h. Consistent with the above results (**Figure 2B**), Pin1 dissociated almost completely from the core particle after heat treatment (**Figure 2D**, top panel; lane 3 vs. 6). To allow the specific antibody to bind to the inside of the core particle on immunoblots, the PVDF membrane was treated with 0.2 N NaOH for 1 min to open up the core particle. It was then UV-crosslinked prior to immunoblotting (Jung et al., 2012; Jung et al., 2014; Saeed et al., 2021). Although heat treatment dissociated Pin1 from the outside of the core particle, it was also detected on the opened-up core particle (**Figure 2D**, second and third panels; lanes 2 and 3 vs. 5 and 6), with no accompanying changes in core particle levels (**Figure 2D**, fourth panel; lanes 2, 3, 5, and 6). This suggests that Pin1 is incorporated into the core particle. Thus, like Par14 and Par17 (Saeed et al., 2021), Pin1 can bind to the outside and the inside of the core particle.

### The HBc S/TP motifs are highly conserved among ten genotypes of human HBV

**Figure 3:**
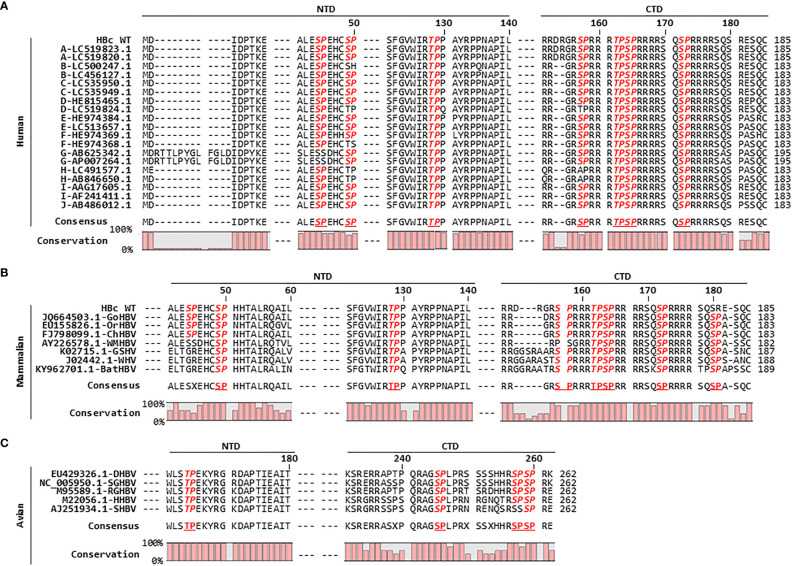
HBc Amino Acid Sequence Alignment Reveals that the HBc of Human, Mammalian, and Avian Hepadnaviruses Harbors Conserved S/TP Motifs. **(A)** The HBc S/TP motifs are highly conserved among ten genotypes of human HBV in the National Center for Biotechnology Information (NCBI), including human HBV [adw subtype (HBc WT)]. The partial HBc NTD and CTD amino acid sequences were aligned using CLC Main Workbench 8 software. The HBV genotype of each isolate, followed by the NCBI accession number, is indicated in the left column. **(B)** Mammalian hepadnavirus HBc S/TP motifs are highly conserved. Alignment of HBc amino acid sequences from gorilla HBV (GoHBV), orangutan HBV (OrHBV), chimpanzee HBV (ChHBV), woolly monkey HBV (WMHBV), ground squirrel hepatitis virus (GSHV), woodchuck hepatitis virus (WHV), and bat HBV (batHBV) is shown. **(C)** Avian hepadnavirus HBc S/TP motifs are highly conserved. Alignment of the HBc amino acid sequences from duck HBV (DHBV), snow goose HBV (SGHBV), ross goose HBV (RGHBV), heron HBV (HHBV), and stork HBV (SHBV) is shown. The accession numbers for the mammalian and avian hepadnaviruses are presented. Conserved S/TP motifs are bold and italicized. The consensus sequences and percentage conservation are shown at the bottom.

**Figure 1** shows the importance of the S/TP motifs within the HBc CTD for the Pin1 interaction (**Figures 1A, E**). Amino acid sequence alignment that HBc ^128^TP within the NTD, and ^162^TP, ^164^SP, and ^172^SP within the CTD, are conserved completely, and that HBc ^44^SP and ^49^SP within the NTD and ^157^SP within the CTD are highly conserved, among 20 isolates of 10 genotypes of human HBc protein (including human HBV [adw subtype (HBc WT)]) in the National Center for Biotechnology Information (NCBI) database (**Figure 3A**). Consistent with the HBc CTD alignment results (**Figure 3A**), previous reports show that ^157^S is less essential for HBV replication (^155^S of ayw subtype) (Lan et al., 1999; Melegari et al., 2005; Lewellyn and Loeb, 2011b; Jung et al., 2014; Nishi et al., 2020), suggesting that the ^162^TP, ^164^SP, and ^172^SP motifs may be more important for the Pin1/core particle interaction. Although HBc NTD seemed not to be important for Pin1/core particle interaction (**Figure 1E**, top panel, lane 3), the completely conserved ^128^TP motif resides within the irregular proline-rich loop 6 (^128^ ***TP*** PAYRPPN^136^) of HBc (**Figure 3A**) (Wynne et al., 1999), suggesting that this motif might play a role in core particle assembly.

HBc amino acid sequence alignment of a further seven isolates of mammalian hepadnaviruses revealed that HBc ^49^SP and ^128^TP within the NTD, and ^162^TP, ^164^SP, ^172^SP, and ^180^SP within the CTD (human HBc WT adw subtype numbering is used for convenience), are completely conserved, and that HBc ^157^SP within the CTD is highly conserved (**Figure 3B**). The HBc of mammalian hepadnaviruses shows a similar S/TP motif pattern to that of 20 isolates of human HBc, with the exception of the first ^44^SP motif in the NTD and the last ^180^SP motif in the CTD. Unlike human HBc, ^44^SP in the NTD is conserved within the HBc amino acid sequences of gorilla HBV (GoHBV), orangutan HBV (OrHBV), and chimpanzee HBV (ChHBV) but not in those of woolly monkey HBV (WMHBV), ground squirrel hepatitis virus (GSHV), woodchuck hepatitis virus (WHV), and bat HBV (batHBV) (**Figure 3B**). The mammalian hepadnavirus HBc ^180^SP motif in the CTD is completely conserved, but human HBc harbors either 11 SR motifs or 9 SP motifs (**Figures 3A, B**). Avian hepadnaviruses HBc ^164^TP in the NTD and ^245^SP and ^259^SP in the CTD are completely conserved, and HBc ^257^SP in the CTD is highly conserved, among five isolates of avian hepadnaviruses (**Figure 3C**). Taken together, we can speculate that the HBc S/TP motifs of orthohepadnaviruses (including human and mammalian hepadnaviruses) and avian hepadnaviruses may play a positive role in replication (**Figure 3**; **Supplementary Figure S1**).

### The HBc ^162^TP, ^164^SP, and ^172^SP motifs are important for the Pin1/core particle interaction

**Figure 4:**
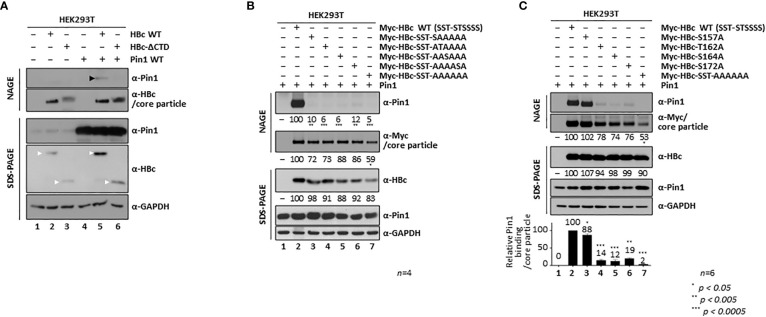
The HBc ^162^TP, ^164^SP, and ^172^SP motifs within the CTD are important for the Pin1/core particle interaction. **(A)** The HBc CTD is important for the Pin1/core particle interaction. HEK293T cells were (co)transfected with mock (lane 1), HBc WT (lane 2), HBc-ΔCTD (lane 3), Pin1 WT (lane 4), HBc WT plus Pin1 WT (lane 5), or HBc-ΔCTD plus Pin1 WT (lane 6). **(B)** A single S/TP phosphoacceptor site within the CTD of HBc is not sufficient for the Pin1/core particle interaction. HEK293T cells were cotransfected with Pin1 WT plus mock (lane 1), Myc-HBc WT (Myc-HBc-SST-STSSSS) (lane 2), Myc-HBc-SST-SAAAAA (lane 3), Myc-HBc-SST-ATAAAA (lane 4), Myc-HBc-SST-AASAAA (lane 5), Myc-HBc-SST-AAAASA (lane 6), or Myc-HBc-SST-AAAAAA (lane 7). **(C)** The HBc ^162^TP, ^164^SP, and ^172^SP motifs within the CTD are important for the Pin1/core particle interaction. HEK293T cells were cotransfected with Pin1 WT plus mock (lane 1), Myc-HBc WT (lane 2), Myc-HBc-S157A (lane 3), Myc-HBc- T162A (lane 4), Myc-HBc-S164A (lane 5), Myc-HBc-S172A (lane 6), or Myc-HBc-SST-AAAAAA (lane 7). The data in the graph represent the mean ± SD of six independent experiments. Two days after transfection, lysates were subjected to NAGE plus immunoblotting, and SDS-PAGE plus immunoblotting **(A-C)**, as described in **Figure 1**. GAPDH was used as a loading control. Representative data are shown. Relative levels of Pin1-bound core particles, core particles, and HBc proteins were calculated using ImageJ 1.50b software. Statistical significance was evaluated using Student’s *t* test. ^*^ *P* < 0.05; ^**^ *P* < 0.005; ^***^ *P* < 0.0005, relative to the corresponding control.

**Figures 1**, **2** show that Pin1 interacts with the core particle through pS/TP motifs in the HBc CTD (Kann and Gerlich, 1994; Kau and Ting, 1998; Daub et al., 2002; Chen et al., 2011; Ludgate et al., 2012; Jung et al., 2014; Nishi et al., 2020). We also examined the CTD-deleted HBc (HBc-ΔCTD) (Jung et al., 2012) by NAGE and immunoblotting to confirm the importance of the HBc CTD for Pin1**/**core particle binding. As expected, Pin1 did not bind to the core particle of HBc-ΔCTD, although the levels of core particles and HBc were comparable with those in HBc WT (**Figure 4A**, second and fourth panels; lane 5 vs. 6), confirming that HBc CTD is important for Pin1 binding.

To identify the S/TP motif(s) important for the Pin1**/**core particle interaction, we constructed HBc mutants that retain only a single phosphoacceptor S/TP site, and in which the corresponding S/TP motifs were substituted with AP motifs by PCR-generated site directed mutagenesis. To discriminate the respective mutants, we denote HBc-WT as bracketed with SST-STSSSS; for example, HBc-SST-AAAAAA (= ^44^S^49^S^128^T-^157^A^162^A^164^A^170^A^172^A^178^A) has sextuple serine and threonine (S/T)-to-alanine (A) mutations at the CTD phosphoacceptor sites and as such is Pin1-binding defective (**Figure 1E**, lane 4; **Figures 4B, C**, lane 7). Even though the ^170^S and ^178^S residues do not belong to an S/TP motif (**Figure 1A**), we included these two sites in the HBc WT and HBc S/T-to-A substituted mutants because both ^170^S and ^178^S are phosphorylated and take part in HBV replication (Jung et al., 2014); thus, these constructs are designated as HBc-WT (SST-STSSSS) and HBc-SST-AAAAAA, instead of HBc-WT (SST-STSS) and HBc-SST-AAAA. Pin1/core particle binding by HBc mutants harboring only a single phosphoacceptor S/TP site was examined by NAGE and immunoblotting (**Figure 4B**). The results showed that the Pin1/core particle interaction was severely impaired by the core particle with only a single S/TP motif on HBc, similar to the Pin binding-defective HBc-SST-AAAAAA mutant (**Figure 4B**; lanes 3−6 vs. lane 7). This suggests that a single S/TP motif on HBc is not sufficient for the Pin1**/**core particle interaction. In addition, the levels of the mutant HBc proteins and the core particles were less than those of the HBc WT and the core particle; also, the level of HBc sextuple mutant and the core particle was the least (**Figure 4B**, second and third panels; lane(s) 2 vs. 3−6 vs. 7). This result was similar to that reported previously for a T160A and S162A double mutant (Nishi et al., 2020).

Next, we constructed single S/T-to-A mutants by PCR-generated site directed mutagenesis (HBc-S157A, HBc-T162A, HBc-S164A, and HBc-S172A); these mutants retain three S/TP motifs in the CTD. Surprisingly, NAGE and immunoblotting showed that the Pin1/core particle interaction was also severely impaired in the HBc-T162A, HBc-S164A, and HBc-S172A mutants but not the HBc-S157A mutant, demonstrating that the ^162^TP, ^164^SP, and ^172^SP motifs are important (**Figure 4C**, top panel; lane 2 vs. lanes 4−6), whereas the ^157^SP motif is less important (**Figure 4C**, top panel; lane 2 vs. 3), for the Pin1**/**core particle interaction. These data are in accordance with amino acid sequence alignment results (**Figure 3**). Unlike a previous report that emphasized the importance of ^162^TP and ^164^SP (^160^TP and ^162^SP) for Pin1/HBc binding (Nishi et al., 2020), we found that the ^162^TP, ^164^SP, and ^172^SP motifs are important (**Figure 4C**). Although the amount of HBc protein by these single S/TP motif mutants was comparable (**Figure 4C**, third panel; lane 2 vs. 3−6), the amount of core particle by HBc-T162A, HBc-S164A, and HBc-S172A mutants was less, although not as significantly less as in the case of the HBc sextuple mutant (**Figure 4C**, second panel; lane 2 vs. 4−6 vs. 7).

Since we constructed HBc mutants harboring S/T-to-A substitutions at the S/TP motifs, the newly generated AP motifs may serve as binding sites for other proline-directed PPIases such as cyclophilins or FKBPs. To examine whether the AP motifs serve as binding sites for cyclophilins or FKBPs, we performed NAGE followed by immunoblotting with anti-cyclophilin A or anti-FKBP12 antibodies (**Supplementary Figure S2**). The result showed that the HBc-SST-AAAAAA mutant does not bind to cyclophilin A or FKBP12.

### HBc NTD S/TP motifs contribute to core particle stability and assembly

**Figure 5:**
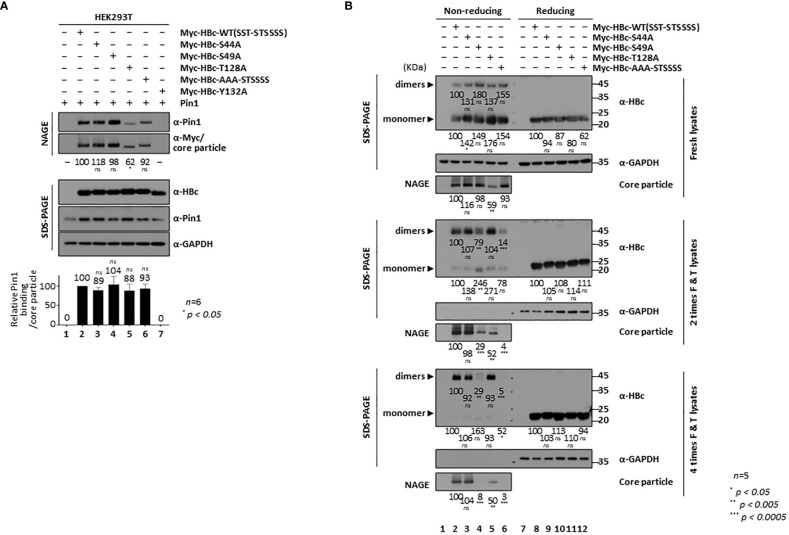
HBc NTD S/TP motifs Contribute to HBV Core Particle Stability and Assembly. **(A)** HBc NTD S/TP motifs are not important for the Pin1/core particle interaction. HEK293T cells were (co)transfected with Pin1 WT plus mock (lane 1), Myc-HBc WT (lane 2), Myc-HBc-S44A (lane 3), Myc-HBc-S49A (lane 4), Myc-HBc-T128A (lane 5), Myc-HBc-AAA-STSSSS (lane 6), or Myc-HBc-Y132A (lane 7). **(B)** HBc NTD S/TP motifs contribute to the structural stability of the HBV core particle. HEK293T cells were transfected with mock (lane 1), Myc-HBc WT (SST-STSSSS) (lane 2), Myc-HBc-S44A (lane 3), Myc-HBc-S49A (lane 4), Myc-HBc-T128A (lane 5), or Myc-HBc-AAA-STSSSS (lane 6). Two days after transfection, lysates were put through repeated freezing and thawing cycles and then subjected to nonreducing PAGE or reducing SDS-PAGE **(B)**. Lysates were subjected to NAGE plus immunoblotting, and SDS-PAGE plus immunoblotting **(A, B)**, as described in **Figure 1**. GAPDH was used as a loading control. Representative data are shown. Relative levels of Pin1-bound core particles, core particles, HBc proteins, and dimeric and monomeric HBc proteins, were calculated using ImageJ 1.50b software. Statistical significance was evaluated using Student’s *t* test. *ns*, not significant; ^*^ *P* < 0.05; ^**^ *P* < 0.005; ^***^ *P* < 0.0005, relative to the corresponding control.

Although we already demonstrated that HBc NTD S/TP motifs are not important for the Pin1**/**core particle interaction (**Figure 1E**, lane 3 vs. 4), we further investigated the HBc NTD single and triple S/T-to-A substitution mutants by NAGE and immunoblotting (**Figure 5A**). We included a particle-defective HBc-Y132A mutant as a negative control. With the exception of the core particles from the Myc-HBc-T128A mutant, the amount of core particles from HBc NTD mutants was comparable with that from HBc-WT (**Figure 5A**, second panel; lane(s) 2−4, 6 vs. 5). Although the core particle level of Myc-HBc-T128A was significantly lower than that of Myc-HBc WT and other HBc NTD mutants, binding of Pin1 binding to the core particles was unaffected (**Figure 5A**, bottom graph; lanes 2 vs. 3−6). Consistent with this, a previous study shows that the completely conserved ^128^TP motif (**Figure 3A**) in an irregular proline-rich loop (Wynne et al., 1999) plays a role in core particle assembly.

To scrutinize the roles of the HBc NTD S/TP motifs in core particle assembly and/or structural stability, we subjected Myc-HBc-WT- and Myc-HBc-NTD S/TP motif mutant-transfected HEK293T cell lysates to repeated freeze/thaw cycles (**Figure 5B**). Expression of HBc monomers was comparable under reducing conditions (**Figure 5B**, right top, right third, right fifth panels; lane(s) 8 vs. 9−12). As shown above (**Figure 5A**, second panel), core particle levels from fresh lysates were comparable (the exception was the core particle of Myc-HBc-T128A) on NAGE and immunoblot analysis (**Figure 5B**, left third panel). Repeated freezing and thawing led to almost complete disassociation of Myc-HBc-S49A and Myc-HBc-AAA-STSSSS core particles (**Figure 5B**, left sixth and ninth panels; lanes 2, 3 vs. 4, 6). Similarly, dimers produced by the Myc-HBc-S49A and Myc-HBc-AAA-STSSSS mutants disappeared after repeated freezing and thawing under nonreducing conditions (**Figure 5B**, seventh panel; lanes 4 and 6). As shown for the Myc-HBc-S49A and Myc-HBc-AAA-STSSSS mutants, the HBc NTD S/TP motifs may play a role in HBV core particle stability in the absence of Pin1-binding activity. In the case of Myc-HBc-T128A (**Figure 5B**, left third panel; lane(s) 5 vs. 3, 4 and 6), the levels of core particle and dimer were not affected by repeated freezing and thawing (**Figure 5B**, lane 5), further supporting the notion that the ^128^TP motif plays a role in core particle assembly. When we cotransfected with HBc-deficient mutant HBV plus HBc WT or HBc NTD S/TP motif mutants, HBc-deficient mutant HBV replication was not rescued well when trans-complemented with HBc NTD S/TP motif mutants such as Myc-HBc-S49A, Myc-HBc-T128A, and Myc-HBc-AAA-STSSSS, especially by Myc-HBc-T128A (**Supplementary Figure S1**). In accordance with above notions, HBc NTD S/TP motifs are important for HBV replication.

### The ^16^Ser and ^34^Trp substrate binding residues of Pin1 increase core particle stability through the Pin1/core particle interaction

**Figure 6:**
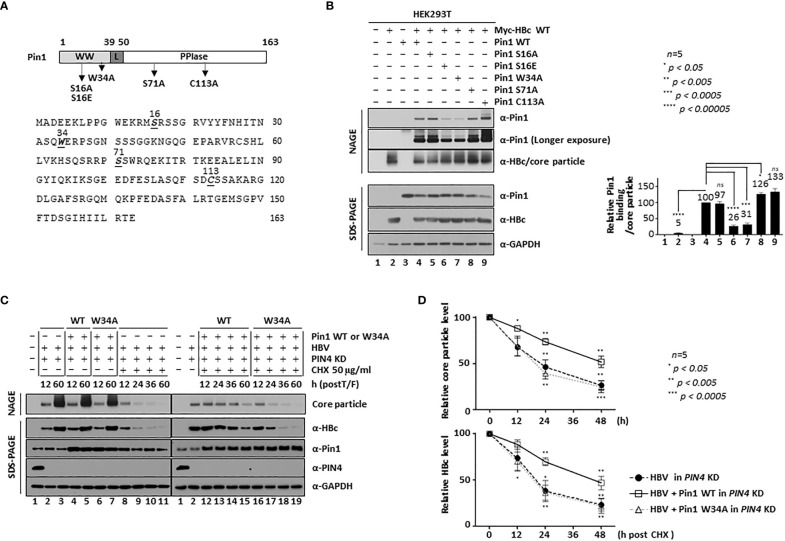
The ^16^Ser and ^34^Trp Substrate Binding Residues of Pin1 Promote Core Particle Stability. **(A)** Schematic diagram and amino acid sequences of Pin1. The Pin1 WW, linker, and PPIase domains are indicated. Important amino acid residues are shown in bold and italics and are underlined. The substrate binding-positive dephosphorylated mimetic S16A, the substrate binding-negative phosphorylated mimetic S16E, the substrate binding-negative W34A, and the PPIase-active S71A and PPIase-inactive C113A mutants are indicated on the diagram. **(B)** The substrate binding ^16^Ser and ^34^Trp residues of Pin1 are important for the Pin1/core particle interaction. HEK293T cells were transfected with mock (lane 1), Myc-HBc WT (lane 2), or Pin1 WT (lane 3) or cotransfected with HBc WT plus Pin1 WT (lane 4), Pin1 S16A (lane 5), Pin1 S16E (lane 6), Pin1 W34A (lane 7), Pin1 S71A (lane 8), or Pin1 C113A (lane 9). **(C, D)** Pin1 WT promotes core particle stability. HEK293T-sh*PIN4* KD cells were (co)transfected with mock (lane 1), HBV WT (lanes 2, 3, 8-11), Pin1 WT plus HBV WT (lanes 4, 5, 12-15), or Pin1 W34A mutant plus HBV WT (lanes 6, 7, 16-19). At 12 h post-transfection, HEK293T cells were treated with 50 μg/ml cycloheximide for 0, 12, 24, or 48 h (lanes 8-19), and lysates were prepared at the indicated times. Lysates were subjected to NAGE plus immunoblotting, and to SDS-PAGE plus immunoblotting **(B, C)**, as described in **Figure 1**. A rabbit monoclonal anti-PIN4 antibody was used to detect Par14/Par17 proteins. GAPDH was used as a loading control. Representative data are shown. Relative levels of Pin1-bound core particles, core particles, and HBc proteins were calculated using ImageJ 1.50b software. Statistical significance was evaluated using Student’s *t* test. *ns*, not significant; ^*^ *P* < 0.05; ^***^ *P* < 0.0005; ^****^ *P* < 0.00005, relative to the corresponding control.

The Pin1 residues that are important for substrate binding and PPIase activity have been identified (Lu and Hunter, 2014; Zhou and Lu, 2016). Indeed, ^16^S and ^71^S act as phosphoacceptors that affect substrate binding and PPIase activity, respectively; tryptophan 34 (^34^Trp: ^34^W) required for substrate binding and cysteine 113 (^113^Cys: ^113^C) required for PPIase activity are indicated in **Figure 6A** (Rippmann et al., 2000; Watashi et al., 2008; Lu and Hunter, 2014). To identify the Pin1 residues involved in HBV core particle binding, we constructed a substrate binding-positive dephosphorylated mimetic S16A mutant, a substrate binding-negative phosphorylated mimetic ^16^S-to-Glu (S16E) mutant, a substrate binding-deficient W34A mutant, a PPIase-active S71A mutant, and a PPIase-inactive C113A mutant (**Figure 6A**). We then examined their ability to bind core particles by conducting NAGE and immunoblotting. As expected, the substrate binding-negative S16E and W34A mutants showed impaired core particle binding, whereas the S16A, S71A, and C113A mutants bound to core particles as efficiently as or more efficiently than Pin1 WT (**Figure 6B**; lane(s) 4 vs. 6, 7 vs. 5, 8, 9). This demonstrates that the substrate binding ^16^S and ^34^W residues of Pin1 are important for the Pin1/core particle interaction. The results for the PPIase-inactive C113A mutant show that PPIase activity is not required for the Pin1/core particle interaction.

HCC and other cancer cells express high levels of Par14/Par17 protein (Saeed et al., 2019) (**Figure 6C**, fourth panel; lane 1) and Par14/Par17 bind to both HBc and the core particle to increase their stability (Saeed et al., 2021); in addition, Par14 might compensate for a lack of Pin1 (Uchida et al., 2003). Therefore, we hypothesized that the effect of Pin1 might be less than optimal in the presence of Par14/Par17. To test this, we transduced HEK293 cells with lentiviral *PIN4* shRNA to generate *PIN4* KD HEK293T cells (pLK0.1-shPIN4#1) (Table) (Saeed et al., 2019). Since Pin1 proteins are involved in the stability of many substrate proteins (Liou et al., 2011; Lu and Hunter, 2014; Zhou and Lu, 2016), we investigated the levels of HBc and core particles in cycloheximide-treated *PIN4* KD HEK293T cells transfected with HBV WT plus mock-, Pin1 WT-, or Pin1 W34A. At 12 h post-transfection, cells were treated with 50 µg/ml of cycloheximide to inhibit protein synthesis (**Figure 6C**; lanes 8−19). In *PIN4* KD cells (**Figure 6C**, fourth panel, lanes 2−19), we detected endogenous Pin1 (third panel; lanes 1−3, 8−10), overexpression of Pin1 WT (third panel; lanes 4, 5, 12−15), and overexpression of the Pin1 W34A mutant (third panel; lanes 6, 7, 16−19). At the same time, we compared HBc protein levels (**Figure 6C**, second panel). At 12 h after cycloheximide treatment (i.e., 24 h post-transfection), there was no significant change in HBc protein levels (**Figure 6C**, second panel; lanes 9, 13, 17; **Figure 6D**, lower panel), confirming that HBc protein is stable. At 24 and 48 h postcycloheximide treatment (i.e., at 36 and 60 h post-transfection), HBc protein levels fell significantly; however, HBc levels in the Pin1 WT-cotransfected cells decreased to a lesser extent than those in mock- or Pin1 W34A mutant-cotransfected cells (**Figure 6C**, second panel; lanes 10, 11 vs. 14, 15 vs. 18, 19; **Figure 6D**, lower panel). Accordingly, in the absence of a new supply of HBc protein, core particle levels in Pin1 WT-overexpressing cells decreased to a lesser extent than those in mock- or Pin1 W34A mutant-cotransfected cells (**Figure 6C**, top panel; lanes 12−15 vs. 8−11 vs. 16−19; **Figure 6D**, upper panel), demonstrating that Pin1 WT, but not the substrate binding negative Pin1 W34A mutant, increases core particle stability, possibly through the Pin1/core particle interaction. Since Pin1 WT alone increased the stability of the core particle, supposedly through the Pin1/core particle interaction, in the absence of *PIN4* (i.e., in *PIN4 KD* cells). Therefore, increasing the stability of the core particles increases the level of HBc proteins (**Figures 6C, D**).

Alternatively, we can hypothesize that Pin1 WT might facilitate core particle assembly. Since Pin1 does not bind to monomeric or dimeric HBc proteins (**Figure 1E**), Pin1 WT may facilitate core particle assembly via a partial intermediate form. This hypothesis is supported by the data in **Figure 2D**.

### Inhibition of parvulin or *PIN1* KD downregulates HBV replication

**Figure 7:**
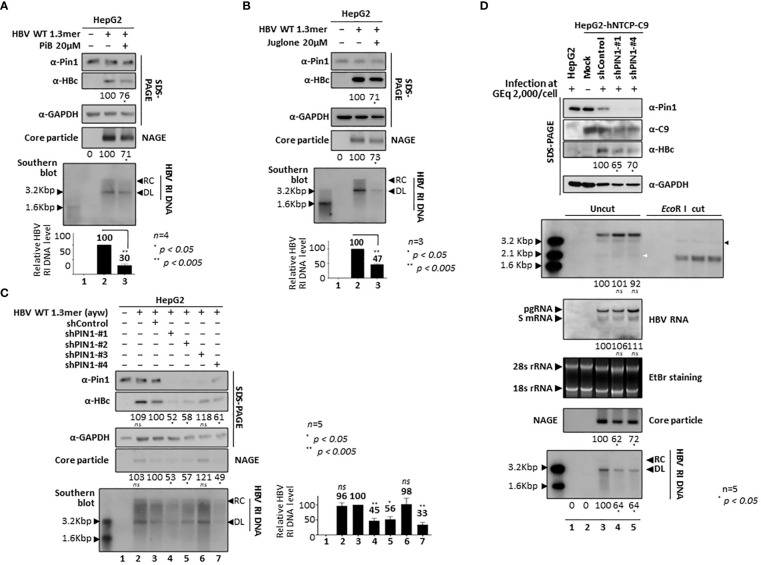
Pin1 Inhibition or *PIN1* KD Downregulates HBV Replication. **(A, B)** Parvulin inhibitors PiB and Juglone downregulate HBV replication. HepG2 cells were transfected with mock (lane 1) or 1.3mer HBV WT (adw) (lanes 2 and 3). At 48 h post-transfection, HepG2 cells were treated with DMSO (lane 2), 20 μM PiB (lane 3) **(A)**, ethanol (lane 2), or 20 μM Juglone (lane 3) **(B)** for 24 h. **(C)** HBV replication is downregulated in *PIN1* KD cells. HepG2 cells were transduced with lentivirus-like particles containing control shRNA (shControl) (lane 3) or *PIN1*-targeting shRNAs (shPIN1-#1, shPIN1-#2, shPIN1-#3, and shPIN1-#4) (lanes 4-7). Nontransduced (lane 2) and transduced (lane 3-7) HepG2 cells were transiently transfected with 1.3mer HBV WT (ayw). Lane 1 shows a negative control without transduction and transfection. At 3 days post-transfection, cell lysates were prepared, and core particle immunoblotting after NAGE and SDS-PAGE plus immunoblotting were performed as described in **Figure 1**. Southern blotting was performed to detect HBV DNA synthesis. In brief, HBV DNA was extracted from isolated core particles, separated, transferred to a nylon membrane, hybridized with a random-primed ^32^P-labeled full-length HBV specific probe, and subjected to autoradiography. HBV replicative intermediate, partially double-stranded relaxed circular, and double-stranded linear DNAs are marked as HBV RI DNA, RC, and DL, respectively **(A-C)**. **(D)** *PIN1* KD decreases HBV replication in infected cells. HepG2 (lane 1), HepG2-hNTCP-C9-shControl (lane 3), HepG2-hNTCP-C9-shPIN1-#1 (lane 4), and HepG2-hNTCP-C9-shPIN1-#4 (lane 5) cells were grown in collagen-coated 6-well plates, infected with 2×10^3^ GEq of HBV per cell, and lysed at 9 days postinfection. Lane 2 represents a mock-infected HepG2-hNTCP-C9 cell. Lysates were subjected to NAGE plus immunoblotting and SDS-PAGE plus immunoblotting, as described in **Figure 1**. A mouse monoclonal anti-C9 antibody was used to detect hNTCP-C9. GAPDH was used as a loading control. Southern blotting was performed to detect HBV DNA synthesis, as described above. For northern blotting, total RNAs were prepared at 5 days postinfection. In brief, 20 μg of total RNA was separated by 1% formaldehyde agarose gel electrophoresis, transferred to nylon membranes, hybridized, and subjected to autoradiography as described above for southern blotting. Next, cccDNA was extracted and subjected to southern blotting without linearization (Uncut) or following linearization with *Eco*R I (*Eco*R I cut). The white and black arrowheads indicate cccDNA and linearized cccDNA to 3.2 kb genome-length, respectively. The 2.1 kb cccDNA, 3.5 kb pgRNA, 2.4 and 2.1 kb S mRNAs, and 28S and 18S rRNAs are indicated. Representative data are shown. Relative levels of RI DNAs, cccDNA, HBV RNAs, core particles, and HBc proteins were calculated using ImageJ 1.50b software. Statistical significance was evaluated using Student’s *t* test. *ns*, not significant; ^*^ *P* < 0.05; ^**^ *P* < 0.005, relative to the corresponding control.

To further examine the function of Pin1 during HBV replication, we used parvulin inhibitors PiB (Diethyl-1,3,6,8-tetrahydro-1,3,6,8-tetraoxobenzo[lmn][3,8]phenanthroline-2,7-diacetic acid; 20 µM in DMSO; CAS 64005-90-9, Calbiochem) (**Figure 7A**) and Juglone (5-hydroxy-1,4-naphthoquinone, 20µM in ethanol, AG17724, Sigma-Aldrich) (**Figure 7B**). At 48 h post-transfection, 1.3mer HBV WT (adw) transfected HepG2 cells were treated with these inhibitors for 24 h. PiB is a competitive reversible inhibitor of parvulin PPIases such as Pin1 and Par14/Par17 (Uchida et al., 2003). Juglone, a natural compound derived from walnut trees, is a competitive irreversible inhibitor that inhibits and/or inactivates members of the parvulin PPIase family; it does not affect other PPIase families (Hennig et al., 1998). Consistent with our previous report (Saeed et al., 2019), we found that HBc protein expression, core particle assembly, and HBV replicative intermediate (RI) DNA synthesis fell significantly after 24 h treatment with PiB (**Figure 7A**; lane 2 vs. 3) and Juglone (**Figure 7B**; lane 2 vs. 3). Unlike our previous study, in which we treated cells with PiB and Juglone for 72 h (Saeed et al., 2019), we exposed cells to PiB and Juglone for 24 h at 48 h post-transfection. The results of these experiments indicate that parvulin proteins affect HBV replication in multiple ways (Saeed et al., 2019; Saeed et al., 2021; Kim, 2023).

To demonstrate that Pin1 affects HBV replication, we generated *PIN1* KD HepG2 cells using a lentiviral shRNA system (Table). Briefly, HepG2 cells were transduced with lentiviral control shRNA (pLK0.1-shControl) or *PIN1* shRNAs (pLK0.1-shPIN1#1−4). Lentivirus-transduced, puromycin-selected *PIN1* KD HepG2 cells were then transfected with 1.3mer ayw HBV WT (**Figure 7C**). HBc protein expression, core particle assembly, and HBV RI DNA synthesis in *PIN1* KD HepG2 cells (**Figure 7C**, top panel; lane 3 vs. 4−7), but not in shPIN1#3-transduced cells, decreased significantly (**Figure 7C**; lanes 4−7), suggesting that Pin1 may support efficient HBV replication.

To examine the effects of Pin1 on HBV infection, we transduced HepG2-hNTCP-C9 cells with lentiviral control shRNA or *PIN1* shRNAs (shPIN1-#1 or #4). Following HBV infection, HBc protein expression, core particle assembly, and HBV RI DNA synthesis decreased significantly upon *PIN1* KD (**Figure 7D**, third, eighth, and bottom panels; lane 3 vs. 4 and 5); however, the levels of HBV pgRNA, subgenomic RNAs, and cccDNA were unaffected (**Figure 7D**, fifth and sixth panels; lane 3 vs. 4 and 5). This result clearly demonstrates that Pin1 affects HBV replication after HBV RNA synthesis.

### Pin1 overexpression upregulates HBV replication

**Figure 8:**
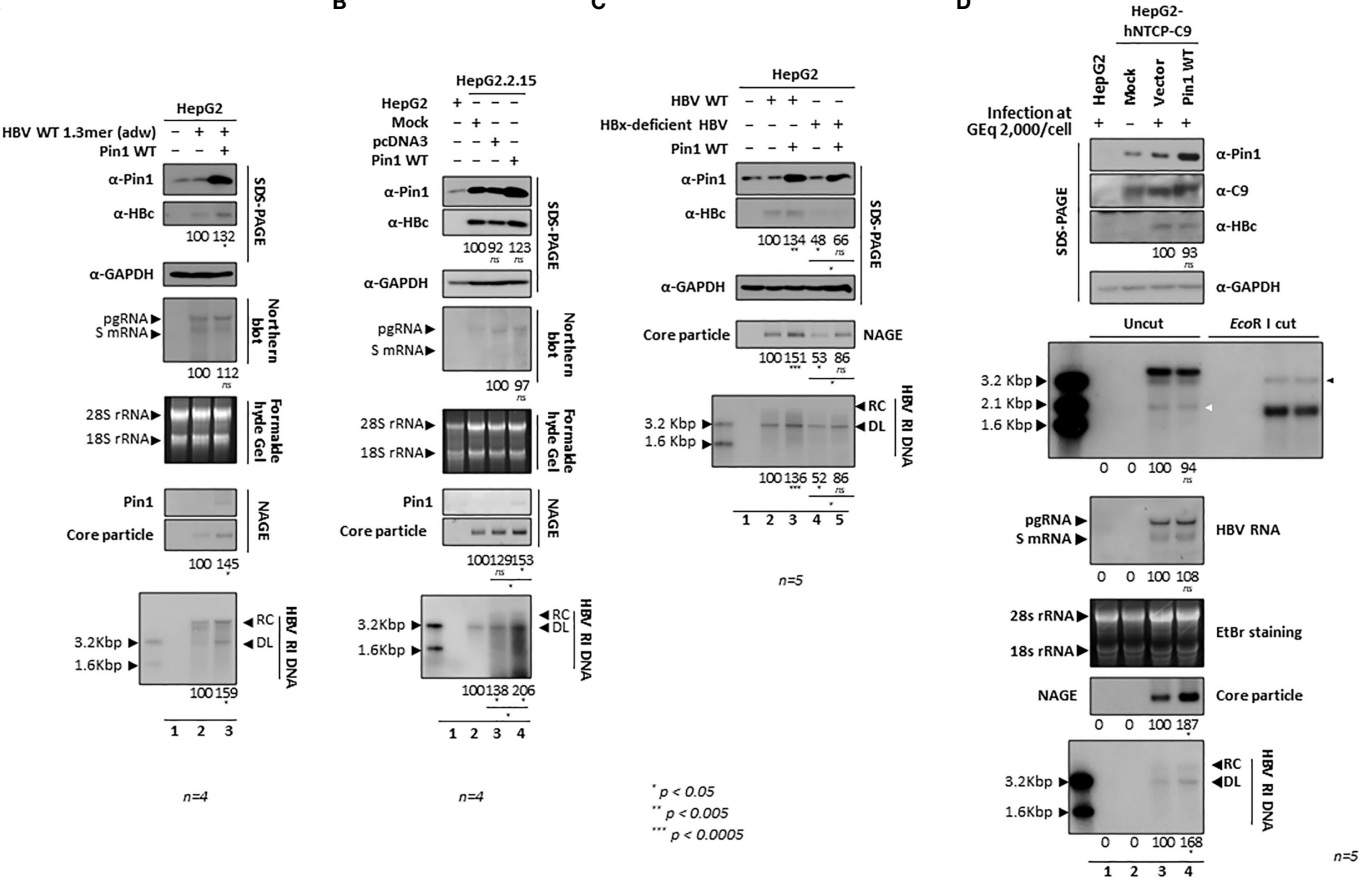
Overexpression of Pin1 Upregulates HBV Replication. **(A)** HBV replication in HepG2 cells is upregulated by overexpression of Pin1. HepG2 cells were transiently (co)transfected with mock (lane 1), 1.3mer HBV WT (adw) (lane 2), or 1.3mer HBV WT (adw) plus Pin1 WT (lane 3). **(B)** HBV replication in HepG2.2.15 cells increases upon overexpression of Pin1. HepG2.2.15 cells were transiently transfected with mock (lane 2), pcDNA3 (lane 3), or Pin1 WT (lane 4). Mock transfected HepG2 cells were used as a negative control (lane 1). **(C)** Upregulated replication of HBV upon overexpression of Pin1 is independent of HBx. HepG2 cells were transiently (co)transfected with mock (lane 1), HBV WT (adw) (lane 2), HBV WT (adw) plus Pin1 WT (lane 3), HBx-deficient HBV mutant (lane 4), or HBx-deficient HBV mutant plus Pin1 WT (lane 5). Transcription of pgRNA from HBV WT and an HBx-deficient HBV mutant was controlled by the CMV IE promoter. **(D)** Increased HBV replication in infected cells overexpressing Pin1. HepG2 cells (lane 1) or HepG2-hNTCP-C9 cells transduced with empty vector (lane 3) or Pin1 WT (lane 4) were grown in collagen-coated 6-well plates and infected with 2×10^3^ GEq of HBV per cell (lanes 1, 3, and 4), as described in **Figure 7D**. Lane 2 shows a mock-infected control. The white and black arrowheads indicate cccDNA and linearized cccDNA to 3.2 kb genome-length, respectively. Cells were lysed at 5 (for total RNA) or 9 days postinfection. **(A-D)** Lysates were subjected to NAGE plus immunoblotting and to SDS-PAGE plus immunoblotting, as described in **Figure 1**. GAPDH was used as a loading control. To detect HBV DNA and HBV RNA, southern blotting and northern blotting were performed, respectively, as described in **Figure 7**. cccDNA was subjected to southern blotting, as described in **Figure 7**. The 2.1 kb cccDNA, 3.5 kb pgRNA, 2.4 and 2.1 kb S mRNAs, and 28S and 18S rRNAs are indicated. Representative data are shown. Relative levels of RI DNAs, cccDNA, HBV RNAs, core particles, and HBc proteins were calculated using ImageJ 1.50b software. Statistical significance was evaluated using Student’s *t* test. *ns*, not significant; ^*^ *P* < 0.05; ^**^ *P* < 0.005; ***P < 0.0005, relative to the corresponding control.

Since HBV replication was downregulated by parvulin inhibitors and *PIN1* KD (**Figure 7**), we further investigated the effects of Pin1 overexpression on the HBV life cycle. When HepG2 cells were cotransfected with Pin1 and 1.3mer HBV WT (adw), HBc protein expression, core particle assembly, and HBV RI DNA synthesis increased significantly (**Figure 8A**, top, second, sixth, bottom panels; lane 2 vs. 3). When Pin1 was transiently transfected into HBV-replicating stable HepG2.2.15 cells, HBV RI DNA synthesis was upregulated (**Figure 8B**; lane 4). Even though pcDNA3-transfected HepG2.2.15 cells (usual mock to adjust transfected DNA amount) showed higher expression of HBV RI DNA than no DNA transfected cells (**Figure 8B**, bottom panel, lane 2 vs. 3), core particle assembly and HBV RI DNA synthesis were upregulated to a greater extent in Pin1-overexpressing cells (**Figure 8B**, second, sixth, bottom panels; lane 2 vs. 3 vs. 4). Unlike the above results (**Figures 1C**, **2A, B**, **8A**), HBc protein expression in HepG2.2.15 cells was not significantly increased by Pin1 overexpression. Consistent with the *PIN1* KD experiment (**Figure 7D**), Pin1 had no significant effect on HBV transcription (**Figures 8A, B**, fourth panel).

Since HBx interacts with Pin1 (Pang et al., 2007) and Par14/Par17 (Saeed et al., 2019), and Par14/Par17 upregulate HBV replication in an HBx-dependent manner (Saeed et al., 2019), it may be that Pin1 overexpression also upregulates HBV replication in an HBx-dependent manner. To examine this, HepG2 cells were cotransfected with Pin1 plus CMV-HBV WT (adw) or CMV-HBx-deficient HBV (adw) (Yoon et al., 2011). Consistent with previous reports (Yoon et al., 2011; Piracha et al., 2018; Kim et al., 2022), we found that replication of HBx-deficient HBV was lower than that of HBV WT (**Figure 8C**; lane 2 vs. 4). Unlike Par14/Par17 (Saeed et al., 2019), overexpression of Pin1 upregulated HBV replication in the absence of HBx (**Figure 8C**; lane 4 vs. 5), demonstrating that Pin1-mediated effects are independent of HBx.

To elucidate the effects of Pin1 overexpression in an HBV infection system, we transduced HepG2-hNTCP-C9 cells with lentiviral vectors pCDH or pCDH-*PIN1*, as described previously (Kim et al., 2022). Following HBV infection of HepG2-hNTCP-C9 cells that had been transduced with the vectors or *PIN1*, we found that Pin1 overexpression increased core particle and HBV RI DNA synthesis significantly (**Figure 8D**, eighth and bottom panels; lane 3 vs. 4), whereas HBc protein levels, cccDNA formation, and HBV pgRNA and subgenomic RNA levels did not change significantly (**Figure 8D**, third, fifth, and sixth panels; lane 3 vs. 4). Consistent with the data for Pin1-overexpressing HepG2.2.15 cells (**Figure 8B**, second panel), HBc levels in HBV-infected HepG2-hNTCP-C9 cells did not increase (**Figure 8D**, third panel). It is extraordinary that core particle levels increased without concomitant increases in pgRNA and HBc protein levels. Usually, protein levels increase along with RNA levels. Since Pin1 cannot interact with HBc, we speculate that Pin1 stabilizes core particles, thereby increasing core particle levels. Consequently, the increase in HBc levels observed on SDS-PAGE may be due to increased stability of the core particle.

### Fewer Pin1 proteins bind to HBV WT core particles than to RT-defective or priming-deficient core particles

**Figure 9:**
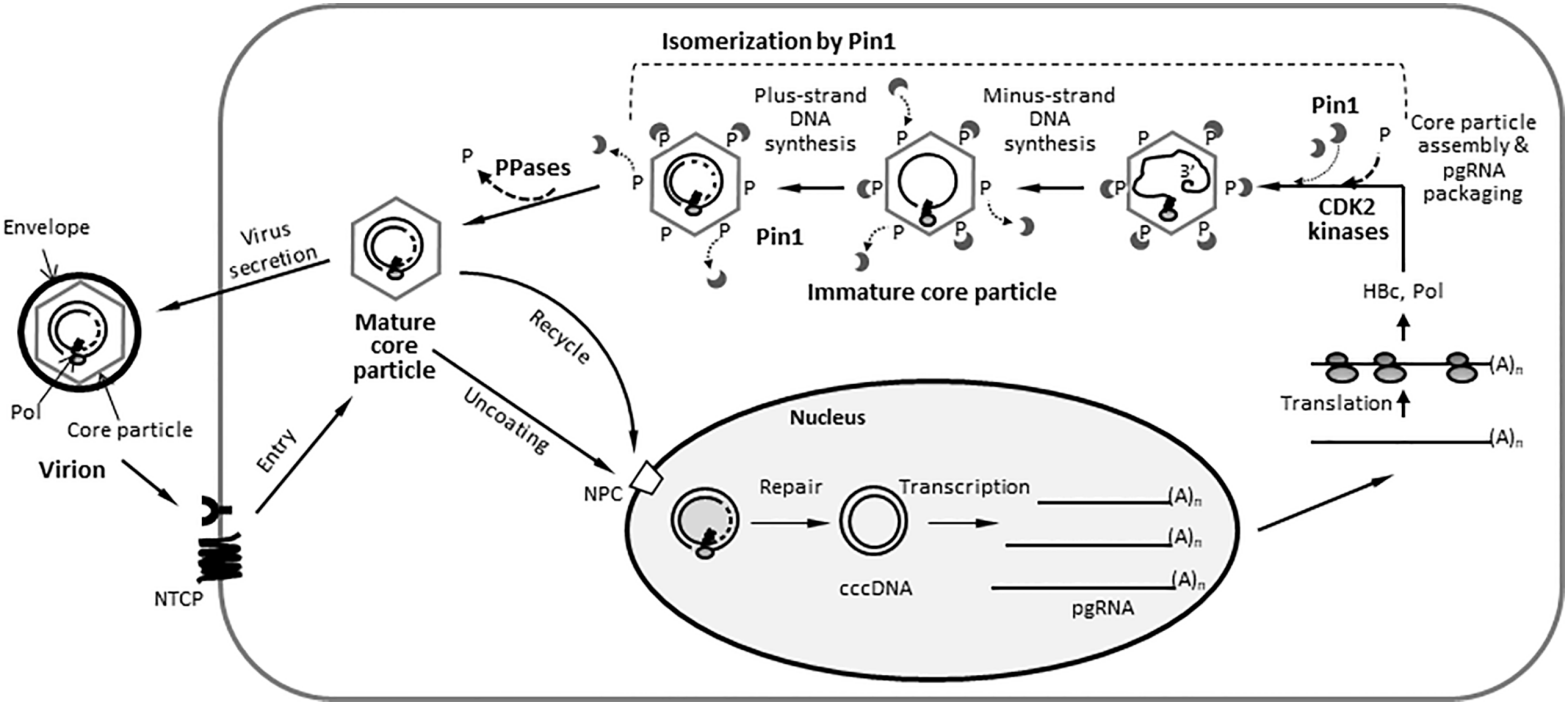
Proposed Model for the Actions of Pin1 During the HBV Life Cycle. After binding to the NTCP receptor on hepatocytes, the HBV virion is internalized and uncoated, and its RC DNA is delivered to the nucleus. The RC DNA genome is repaired to form cccDNA, which serves as the template for transcription. Viral RNAs, including pgRNA, are exported to the cytoplasm, where viral proteins such as HBc and Pol are translated. The pgRNA is copackaged with Pol into immature core particles composed of HBc proteins. At this stage, host CDK2 is supposed to phosphorylate the phosphoacceptor S/TP motifs on HBc, thereby forming Pin1-binding sites on the core particle. The pgRNA is reverse transcribed to minus-strand DNA and then plus-strand DNA, thus forming RC DNA-containing mature core particles. The mature core particles are dephosphorylated via bound Pin1 at S/TP sites on core particle by unidentified host PPases, such as PDP2, followed by envelopment by viral HBs envelope proteins. They are then secreted extracellularly as virions or recycled back to the nucleus to amplify the cccDNA pool. RC DNA, relaxed circular DNA; cccDNA, covalently closed circular DNA; pgRNA, pregenomic RNA; Pol, HBV DNA polymerase; CDK2, cyclin-dependent protein kinase 2; P, phosphates; PPase, host protein phosphatase; PDP2, pyruvate dehydrogenase phosphatase 2.

Immature core particles are heavily phosphorylated by series of kinases, including proline-directed serine/threonine kinases, CDK2, and/or a CDK2-like kinase, which phosphorylate S/TP motifs (Ludgate et al., 2012). Also, mature core particles are dephosphorylated by as-yet-unidentified phosphatases, although protein phosphatase 2A (PP2A) (Ning et al., 2017) or PDP2 (Nishi et al., 2020) have been suggested. Since we demonstrated that Pin1 interacts physically with core particles but not with HBc monomers and dimers (**Figures 1E, F**), we hypothesized that Pin1 may function in this dephosphorylation process by binding to pS/TP on core particles, thereby catalyzing isomerization and conformational changes that facilitate dephosphorylation of these pS/TP motifs; it may then detach from the mature core particles prior to budding or recycling (**Figure 9**).

**Figure 10:**
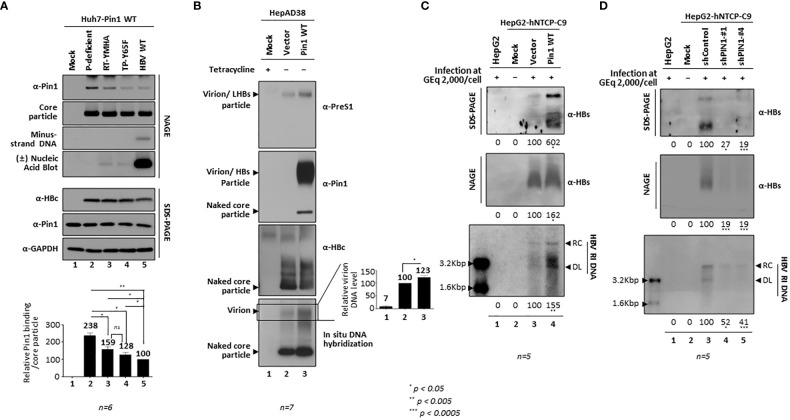
Binding of Pin1 to Intracellular Core Particles Differs According to Replication Stage and Increases Secretion of Virions. **(A)** Fewer Pin1 proteins bind to the mature core particle than to the immature core particle. Stable Huh7-Pin1-expressing cells were transiently transfected with mock (lane 1), the P-deficient mutant (lane 2), the RT-YMHA mutant (lane 3), the TP-Y65F mutant (lane 4), or HBV WT (lane 5). As described in **Figure 1**, lysates were subjected to NAGE plus immunoblotting and to SDS-PAGE plus immunoblotting. *In situ* DNA hybridization was performed to detect minus-strand DNA in the core particle, and *in situ* nucleic acid hybridization was performed to detect plus- and minus-stranded nucleic acids in the core particle. In brief, isolated core particles on a PVDF membrane were treated with 0.2 N NaOH, hybridized to either a DIG-labeled full-length plus-strand HBV RNA probe or a random-primed ^32^P-labeled full-length HBV specific probe, and subjected to autoradiography. **(B)** Pin1 overexpression increases virion secretion in stable HepAD38 cells. HepAD38 cells were transduced with empty vector (lane 2) or with Pin1 (lane 3) transcript-containing pseudoviral particles and then selected with puromycin. After removal of tetracycline to induce HBV transcription, the HepAD38-vector control and HepAD38-Pin1 stable cells were incubated for 3 days, and culture supernatants were harvested. HepAD38-Pin1 stable cells cultured with tetracycline (lane 1) were used as a negative control. Cleared culture supernatants were precipitated by ultracentrifugation on a 20% (w/w) sucrose cushion (26,000 rpm for 3 h at 4°C). Pellets containing HBV virions, subviral particles, and naked core particles were resuspended in TNE buffer and subjected to NAGE plus immunoblotting or *in situ* nucleic acid blotting to detect virions and/or LHBs particles, Pin1 on virions and/or on naked core particles, naked core particles, or virion DNA. **(C)** Pin1 overexpression increases virion secretion by HBV-infected cells. HepG2 (lane 1), vector-transduced HepG2-hNTCP-C9 (lane 3), and Pin1-transduced HepG2-hNTCP-C9 (lane 4) cells were infected with HBV as described in **Figure 7D**. Lane 2 shows mock-infected HepG2-hNTCP-C9 cells. **(D)** Pin1 KD downregulates secretion of HBV virions from HBV-infected cells. HepG2 (lane 1), shControl-transduced (lane 3), shPIN1-#1-transduced (lane 4), and shPIN1-#4-transduced (lane 5) HepG2-hNTCP-C9 cells were infected with HBV as described. Lane 2 shows mock-infected HepG2-hNTCP-C9 cells. Pellets obtained from culture supernatants were subjected to NAGE plus immunoblotting, SDS-PAGE plus immunoblotting, and southern blotting **(C, D)**, as described in **Figures 1**, **7**. The data in the graphs represent the mean ± SD from six **(A)** or seven **(B)** independent experiments. Representative data are shown. Relative levels of Pin1-bound core particles, virion DNAs, virion HBs proteins, and virions and/or subviral particles were calculated using ImageJ 1.50b software. Statistical significance was evaluated using Student’s *t* test. *ns*, not significant; ^*^ *P* < 0.05; ^**^ *P* < 0.005; ^***^ *P* < 0.0005, relative to the corresponding control.

To test this hypothesis, we examined binding of Pin1 to the core particle at different stages of the HBV life cycle. We examined this in stable Pin1-overexpressing Huh7 cells transiently transfected with a P-deficient mutant harboring empty core particles, a reverse transcription-defective RT-YMHA mutant, the oligomeric minus-strand translocation-defective priming-deficient TP-Y65F mutant (Kim et al., 2004), and HBV WT (**Figure 10A**). When we compared Pin1 binding to immature core particles from the RT-YMHA mutant- and TP-Y65F mutant-transfected cells (Kim et al., 2004) with that to core particles from HBV WT-transfected cells, we found that Pin1 bound more efficiently to immature core particles from RT-YMHA and TP-Y65F mutants than to those from HBV WT (**Figure 10A**, top and bottom panels; lane 5 vs. 3 and 4). Core particles from the P-deficient mutant, supposedly harboring empty core particles, bound the most Pin1 (**Figure 10A**, top and bottom panels; lane 2 vs. 3−5), and core particles from HBV WT bound the least Pin1 (**Figure 10A**, top and bottom panels; lane 5 vs. 2−4). More Pin1 proteins seem to bind to core particles from the RT-YMHA mutant than from the TP-Y65F mutant; however, the difference was insignificant (**Figure 10A**, top and bottom panels; lane 3 vs. 4). Of note, in HBV WT-transfected cells, core particles at multiple replicative stages (immature through the mature) were present. As expected, *in situ* DNA hybridization to detect minus-strand DNA in core particles showed that minus-strand DNA was present only in HBV WT core particles (**Figure 10A**, third panel; lane 5). As expected, *in situ* nucleic acid hybridization detected encapsidated pgRNAs from RT-YMHA and TP-Y65F mutants weakly but detected encapsidated pgRNAs and RI DNAs from HBV WT strongly (**Figure 10A**, fourth panel; lane(s) 3, 4 vs. 5). This indicates that our Pin1/core particle interaction model based on HBV replication stage is plausible (**Figure 9**).

### Pin1 overexpression increases secretion of virions, whereas *PIN1* KD decreases secretion of virions

To further extend this model, we examined culture supernatants from Pin1 WT- and control empty vector-transduced HepAD38 cells by NAGE, followed by immunoblotting with anti-PreS1, anti-Pin1, and anti-HBc antibodies and *in situ* DNA hybridization (**Figure 10B**). More virions and subviral particles containing HBs (LHBs, MHBs, and SHBs) were released, thus the more virion DNA and PreS1 were detected, in Pin1 WT stable cells than control cells (**Figure 10B**, top and bottom panels; lane 2 vs. 3). The anti-Pin1 antibody reacted strongly with virions and subviral particles from Pin1 stable cells (**Figure 10B**, second panel; lane 3), indicating that HBs in virions and subviral particles may also be binding substrates of Pin1. Consistent with this, amino acid sequence analysis of HBs from ten genotypes of human HBV revealed that HBs proteins (including LHBs, MHBs, and SHBs) harbor seven conserved S/TP motifs (according to the amino acid sequence of LHBs in the NCBI database) (**Supplementary Figure S3**). Consistent with the data in **Figure 10B**, Pin1-overexpressing, HBV-infected HepG2-hNTCP-C9 cells showed increased secretion of virions and subviral particles, as demonstrated by increased levels of extracellular HBs and virion DNA (**Figure 10C**; lane 3 vs. 4). By contrast, fewer extracellular HBs and virion DNA were detected in HBV-infected *PIN1* KD cells (**Figure 10D**; lane 3 vs. 4 and 5). Pin1 upregulates HBV RI DNA synthesis as well as secretion of virions and subviral particles. Taken together, we propose that the Pin1/core particle interaction facilitates assembly of HBV core particles, stabilizes them, helps them to mature during the intracellular HBV replicative stage, and facilitates virion secretion (**Figure 9**).

## Discussion

Here, we demonstrate that Pin1 interacts with the HBV core particle but not with dimeric or monomeric HBc protein (**Figure 1**). To date, all identified binding partners of Pin1 are a single protein, not a multiprotein complex (Liou et al., 2011; Lu and Hunter, 2014; Zhou and Lu, 2016). This is the first study to show directly that Pin1 binds to a multiprotein complex comprising the 180 or 240 HBc proteins and is involved in HBV propagation through a Pin1/core particle interaction. Although a previous study showed that the HIV-1 core may bind to Pin1 through the p^16^SP motif on capsid protein (CA) to facilitate uncoating (Misumi et al., 2010), it did not show that a multiprotein complex such as the HIV-1 core, rather than the free CA, is the sole binding partner of Pin1 (Misumi et al., 2010).

When we overexpressed Pin1, the amounts of HBc and the HBV core particle increased (**Figures 1B, C**, **2A, B**, **6C, D**, **8**). Thus, we speculate that Pin1 promotes core particle assembly (**Figure 5**) and can stabilize the core particle and HBc via Pin1/core particle interactions (**Figures 6C, D**). Since dimeric and monomeric HBc proteins did not bind to Pin1 (**Figures 1E**, **D**, **5A**) or to Pin1 incorporated inside the core particle (**Figure 2D**), we hypothesize that the intermediate particulate form may be phosphorylated on S/TP motifs, and that subsequent core particle assembly may be facilitated by Pin1. Even though the HBc NTD S/TP motif is not important for its interaction with Pin1, ^49^SP and ^128^TP are important for particle stability and assembly, respectively (**Figure 5**). Additionally, fewer HBc CTD S/TP motif mutants, especially the sextuple mutant, were assembled at low level (**Figures 4B, C**, lane 7). More remarkably, all seven S/TP motif-substituted mutants, and Myc-HBc-AAA-AAASAS and Myc-HBc-AAA-AAAAAA, were defective in core particle assembly (**Figure 1F**), indicating that HBc S/TP motifs affect core particle assembly.

Previously, we reported that the Par14/Par17-binding ^133^RP motif is in the proline-rich loop (^128^ ***TP*** PA **Y *RP*** PN^136^) involved in the dimer-dimer interaction that facilitates assembly of the core particle (Wynne et al., 1999; Saeed et al., 2021). Of note, ^132^Y is important for the interdimeric interaction (Bourne et al., 2009). Here, we found that the ^128^TP motif (**Figure 3**) also resides in this proline-rich loop, suggesting a role in core particle assembly.

Par14/Par17 dissociate from the core particle upon heat treatment (Saeed et al., 2021). In accordance with this, we found that the Pin1/core particle interaction was weakened significantly, even after heating for 5 min at 65°C, with no reduction in core particle levels (**Figure 2B**). Like APOBEC3G-Pin1 binding in HIV-1 replicating cell lysates (Watashi et al., 2008), we found that a high salt solution weakened Pin1/core particle binding significantly, without reducing core particle levels (**Figure 2A**). Furthermore, the Pin1/core particle interaction *in vitro* was abolished by CIAP treatment (**Figure 2C**); indeed, CIAP dissociates HIV-1 WT cores and reduces Pin1 binding (Misumi et al., 2010). Like other known Pin1 substrate, such as HBx and HIV core (Pang et al., 2007; Misumi et al., 2010; Liou et al., 2011; Lu and Hunter, 2014; Zhou and Lu, 2016), Pin1 binds to HBV phosphorylated core particles, which is inversely associated with replication stage (**Figures 9**, **10A**).

In addition to HBx (Pang et al., 2007) and HBc (Nishi et al., 2020), several viral proteins act as binding substrates for Pin1 to increase viral replication and oncogenesis. These include human T-cell leukemia virus type 1 (HTLV-1) Tax, which increases oncogenesis (Jeong et al., 2009); human immunodeficiency virus type-1 (HIV-1) integrase, APOBEC3G, and CA core protein, all of which promote viral genome integration and replication (Watashi et al., 2008; Manganaro et al., 2010; Misumi et al., 2010); the Epstein-Barr virus DNA polymerase catalytic subunit, which modulates productive viral DNA replication (Narita et al., 2013); the human herpesvirus 8 lytic switch protein Rta, which regulates reactivation (Guito et al., 2014); hepatitis C virus (HCV) NS5A/NS5B proteins, which promote HCV propagation (Lim et al., 2011); and SARS-CoV2 RNA synthesis promotion (Yamamotoya et al., 2021).

The results of our previous studies led us to propose a model in which the HBV core particle/Pin1 interaction may begin at the time of core particle assembly (**Figures 2D**, **5**, **9**), followed by phosphorylation by CDK2 or a CDK2-like kinase (Ludgate et al., 2012). Thus, CDK2 or a CDK2-like kinase can be incorporated into the core particle (Ludgate et al., 2012). During the HBV replication processes, HBV pgRNA encapsidation, minus-strand DNA synthesis, and RC DNA synthesis occur. During the process of maturation, HBV core particles containing RC DNA are completely dephosphorylated, supposedly by the putative HBc phosphatase PDP2 (Nishi et al., 2020). Then, Pin1 dissociates from the mature core particle (**Figure 10A**), which triggers envelopment and secretion (**Figure 9**) (Perlman et al., 2005; Basagoudanavar et al., 2007; Ning et al., 2017).

Since Pin1 interacts with the HBV core particle, the effects of Pin1 on HBV replication should occur at the time of, or shortly after, core particle assembly. Accordingly, we found that overexpression of Pin1 stabilized the core particles (**Figures 6C, D**), increased RI DNA synthesis (**Figure 8**), and increased virion secretion (**Figures 10B, C**) without any changes in the level of HBV RNA transcription (**Figures 8A, B, D**). In the case of Pin1 inhibition or *PIN1* KD, HBV levels decreased along with HBc, core particle, and RI DNA levels (**Figure 7**), without any changes in HBV RNA transcription (**Figure 7D**). The exception was HBc, levels of which were decreased by Pin1 inhibition or *PIN1* KD, possibly via degradation through the lysosomal pathway (Nishi et al., 2020). More than 50% of HCC cases occur in individuals with a chronic HBV infection (El-Serag, 2012); however, the underlying mechanisms are unclear. Multiple factors such as necroinflammation, HBV itself, HBx, and/or the interaction with host proteins may play a role (Pang et al., 2007; Feitelson et al., 2009; Neuveut et al., 2010; Ringelhan et al., 2015); however, HBx is implicated as an HBV oncoprotein (Feitelson et al., 2009; Neuveut et al., 2010; Ringelhan et al., 2015), and the Pin1/HBx interaction increases hepatocarcinogenesis (Pang et al., 2007). Since higher HBV DNA and HBsAg levels increase the risk of hepatocarcinogenesis (Yu et al., 2005; Chen et al., 2006; Kawanaka et al., 2014), our results further strengthen the notion that Pin1 plays a role in HBV-associated hepatocarcinogenesis by interacting with core particles, although we do not exclude involvement of HBx. Since Pin1 has been suggested as a therapeutic cancer drug target (Zhou and Lu, 2016), and development of more potent and specific Pin1 inhibitors is underway, our data suggest that Pin1-targeting drugs may also be good candidate treatments for chronic HBV and HBV-associated HCC.

## Data availability statement

The datasets presented in this study can be found in online repositories. The names of the repository/repositories and accession number(s) can be found in the article/**Supplementary Material**.

## Author contributions

KK – study concept and design, study supervision, data analysis and interpretation, acquisition of funding, drafting the manuscript, and critical revision of the manuscript. HK – data acquisition and interpretation, drafting of the manuscript, statistical analysis. JK, CS, MS, JH, and JJ – administrative, technical, and material support. SP and HS – critical revision of the manuscript. All authors contributed to the article and approved the submitted version.
