# Supplementary material for: Peptidyl-prolyl cis/trans isomerase Pin1 interacts with hepatitis B virus core particle, but not with HBc protein, to promote HBV replication
Source: Front Cell Infect Microbiol. 2023 Jun 19;13:1195063. doi: 10.3389/fcimb.2023.1195063 (PMC10315659; doi:10.3389/fcimb.2023.1195063)
Supplement: Supplementary file 1 [file DataSheet_1.docx]

Supplementary Material

Peptidyl-Prolyl cis/trans Isomerase Pin1 Interacts with Hepatitis B Virus Core Particle, But Not with HBc Protein, to Promote HBV Replication

**Hyeonjoong Kwon*, Jumi Kim, Chanho Song, Muhammad Azhar Sajjad, Jiseon Ha, Jaesung Jung, Sun Park, and Ho-Joon Shin**

*** Correspondence:** Kyongmin Kim: kimkm@ajou.ac.kr

**
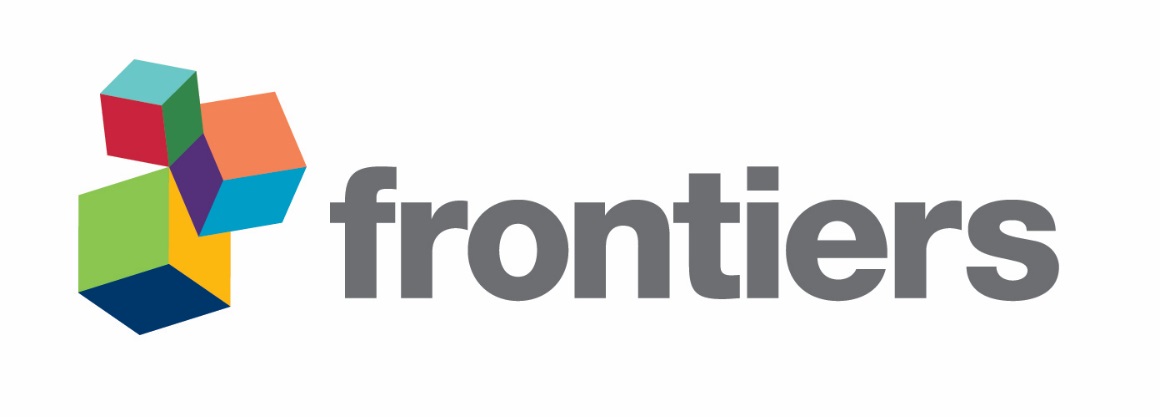
**

**Supplementary Figure S1.**


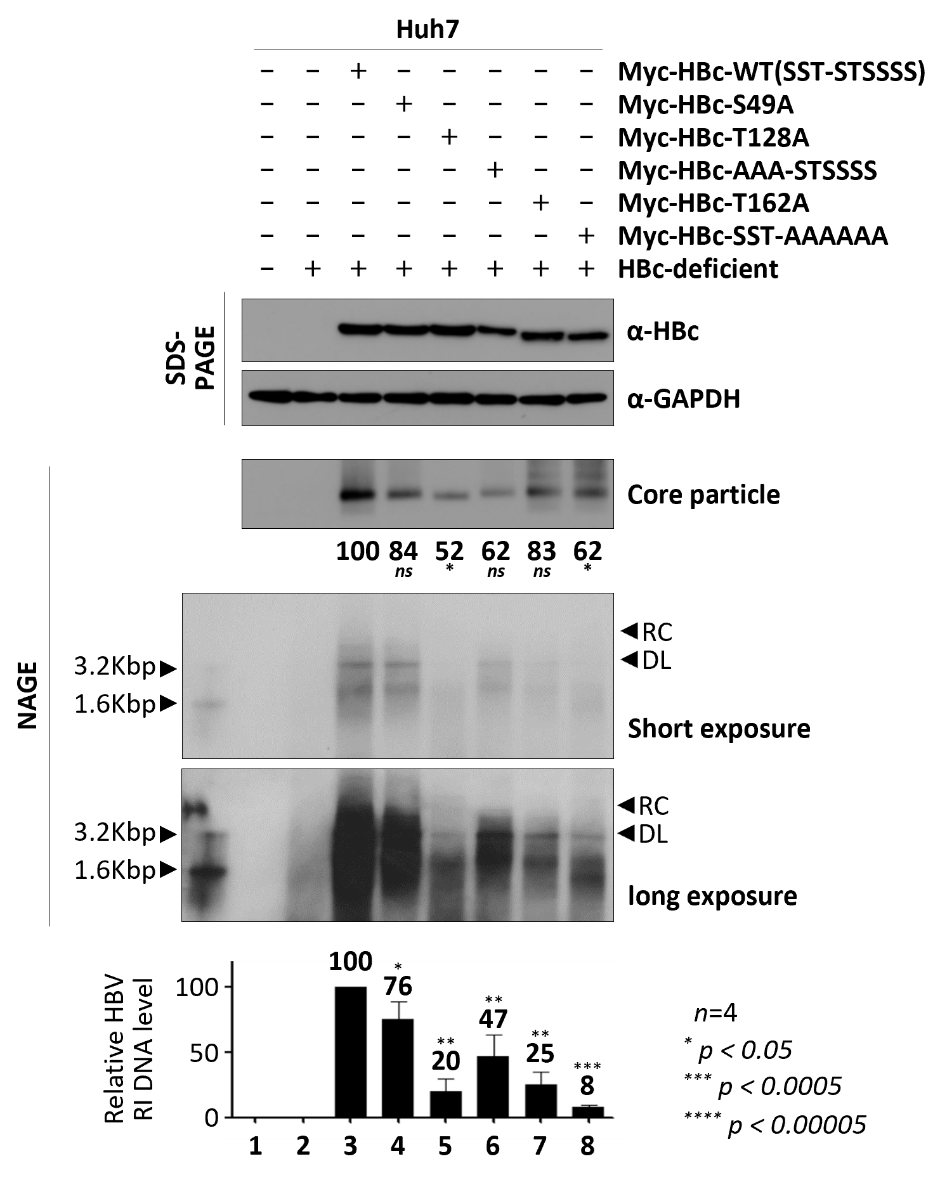


**Supplementary Figure S1. Unlike trans-complementation by HBc WT, that by HBc NTD S/TP mutant proteins, HBc-S49A, HBc-T128A, and HBc-AAA, decrease HBc-deficient mutant HBV replication.** Huh7 cells were (co)transfected with mock (lane 1) and HBc-deficient mutant HBV plus pCMV10-Myc (lane 2), Myc-HBc-WT (lane 3), Myc-HBc-S49A mutant (lane 4), Myc-HBc-T128A mutant (lane 5), Myc-HBc-AAA-STAAAA mutant (lane 6), Myc-HBc-T162A mutant (lane 7), and Myc-HBc-SST-AAAAAA mutant (lane 8), and harvested at 72 h post-transfection. The lysates were subjected to SDS-PAGE plus immunoblotting and NAGE plus immunoblotting as described in the Materials and Methods. HBV DNA synthesis was analyzed by southern blotting. Relative levels of RI DNAs and core particles were calculated using the ImageJ 1.50b software. Statistical significance was evaluated using Student’s *t* test. *ns*, not significant; *, *P* < 0.05; **, *P* < 0.005, ***, *P* < 0.0005, relative to the corresponding control.

**Supplementary Figure S2.**

**
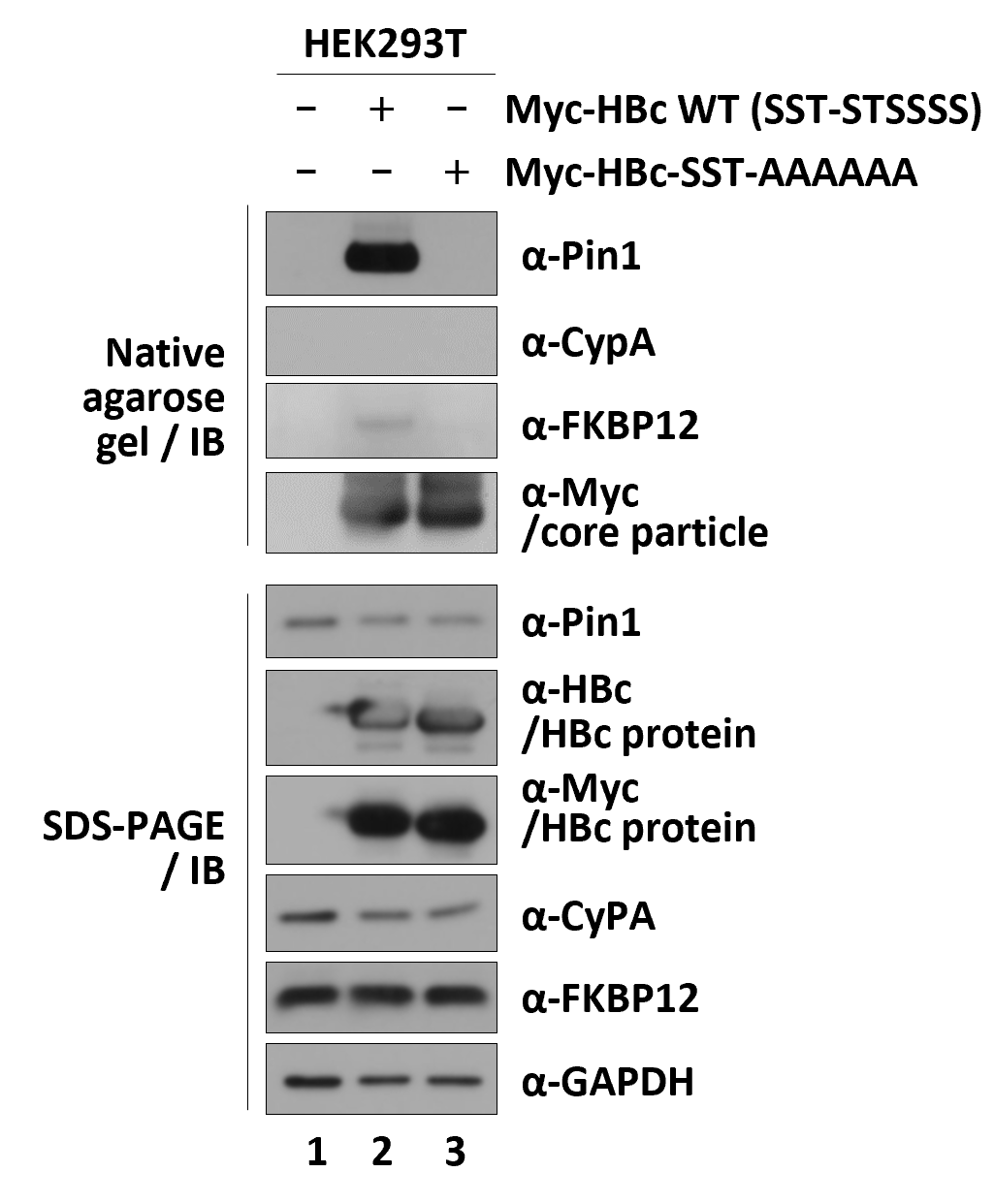
**

**Supplementary Figure S2. AP motifs from HBc-SST-AAAAAA mutant do not bind other PPIases.** HEK293T cells were transfected with mock (lane 1), Myc-HBc WT (lane 2), or Myc-HBc-SST-AAAAAA (lane 3) and harvested at 48 h post-transfection. The lysates were subjected to SDS-PAGE plus immunoblotting and NAGE plus immunoblotting as described in the Materials and Methods. GAPDH was used as a loading control.

**Supplementary Figure S3.**


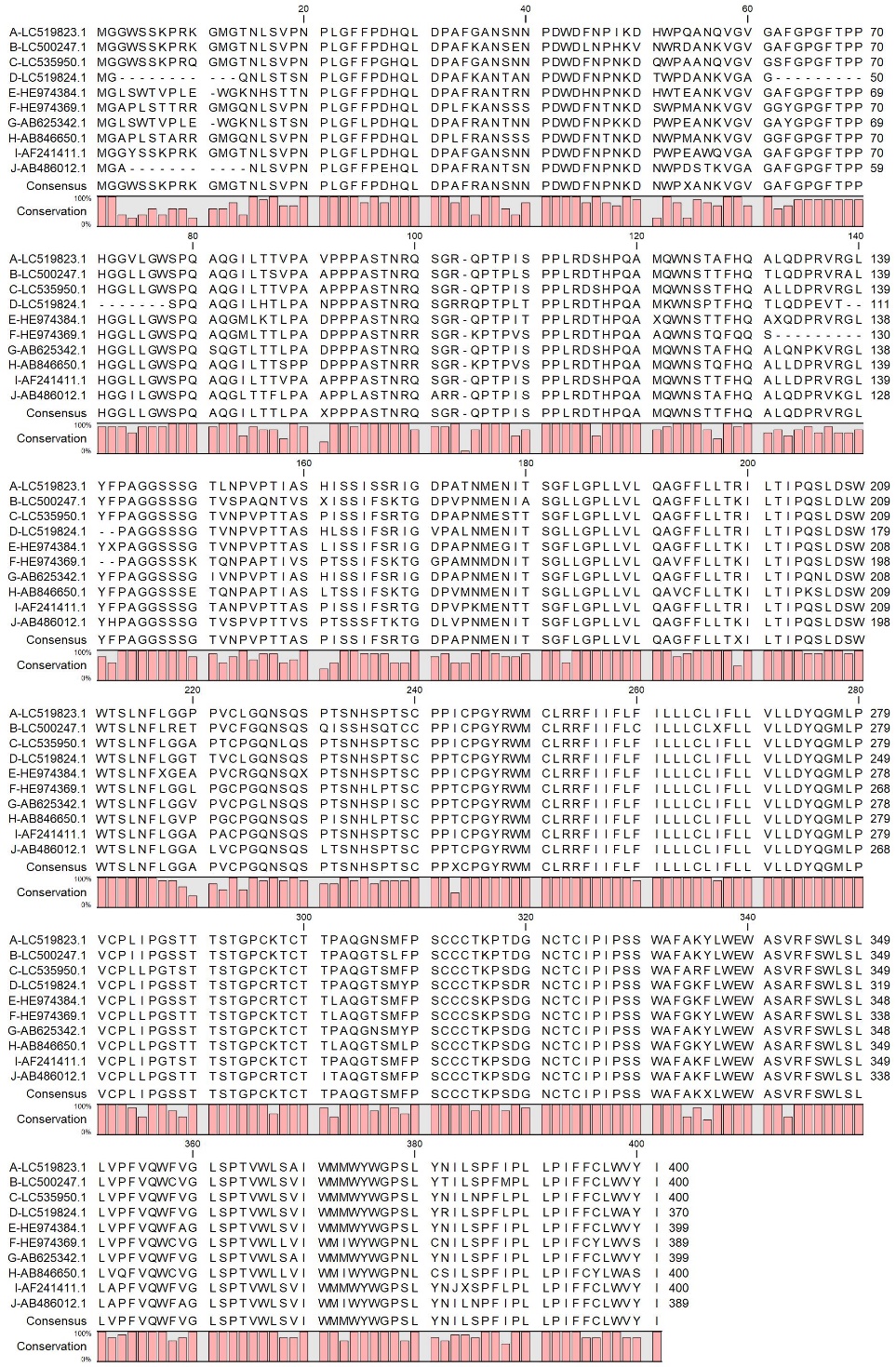


**Supplementary Figure S3. LHBs Amino Acid Sequence Alignment Reveals that Human Hepadnavirus Harbors Conserved S/TP Motifs.** The LHBs S/TP motifs harbor three completely (marked red) and four highly (marked blue) conserved S/TP motifs, among ten genotypes of human HBV in the National Center for Biotechnology Information (NCBI). The amino acid sequences were aligned using CLC Main Workbench 8 software. Each HBV genotype, followed by the NCBI accession number, is indicated in the left column. The consensus sequences and percentage conservation are shown at the bottom.
