# Supplementary material for: Peptidyl-prolyl cis/trans isomerase Pin1 interacts with hepatitis B virus core particle, but not with HBc protein, to promote HBV replication
Source: Front Cell Infect Microbiol. 2023 Jun 19;13:1195063. doi: 10.3389/fcimb.2023.1195063 (PMC10315659; doi:10.3389/fcimb.2023.1195063)
Supplement: Supplementary file 2 [file Table_1.docx]

**Table. Sequences of Primers Used to Construct the Pin1 WT and Mutants and the HBc WT and Mutants, and the Sequences of Pin1 Short Hairpin RNA**

| Plasmid Name | |  | Sequence (5’-3’)^a^ |
| --- | --- | --- | --- |
| Pin1 WT and mutants | pcDNA3-Pin1 WT | F | CAGAG aagcttATGGCGGACGAGGAGAAG^b^ |
|  |  | R | AACTA ggatccTCACTCAGTGCGGAGGATG^c^ |
|  | pcDNA3-Pin1 S16A | F | GGAGAAGCGCATGGCCCGCAGCTCAGGC |
|  |  | R | GCCTGAGCTGCGGGCCATGCGCTTCTCC |
|  | pcDNA3-Pin1 S16E | F | GGAGAAGCGCATGGAACGCAGCTCAGGC |
|  |  | R | GCCTGAGCTGCGTTCCATGCGCTTCTCC |
|  | pcDNA3-Pin1 W34A | F | AACGCCAGCCAGGCTGAGCGGCCCAGC |
|  |  | R | GCTCCCCCGCTCAGCCTGGCTGGCGTT |
|  | pcDNA3-Pin1 S71A | F | CACGGCGGCCCGCGTCCTGGCGGCA |
|  |  | R | TGCCGCCAGGACGCGGGCCGCCGTG |
|  | pcDNA3-Pin1 C113A | F | TCACAGTTCAGCGACGCCAGCTCAGCCAAGGC |
|  |  | R | GCCTTGGCTGAGCTGGCGTCGCTGAACTGTGA |
| HBc WT and mutants | pCMV-Myc-HBc WT | F | CTTAtggccaCGGACATTGACCCT^d^ |
|  |  | R | ATTggtaccCTAACATTGAGATT^e^ |
|  | pCMV-Myc-HBc S44A | F | AAGCCTTAGAGGCTCCTGAGCATTG |
|  |  | R | CAATGCTCAGGAGCCTCTAAGGCTT |
|  | pCMV-Myc-HBc S49A | F | CTGAGCATTGCGCACCTCACCATAC |
|  |  | R | GTATGGTGAGGTGCGCAATGCTCAG |
|  | pCMV-Myc-HBc T128A | F | TGTGGATTCGCGCTCCTCCAGCCTA |
|  |  | R | TAGGCTGGAGGAGCGCGAATCCACA |
|  | pCMV-Myc-HBc S157A | F | ACCGAGGCAGGGCCCCTAGAAGAAG |
|  |  | R | CTTCTTCTAGGGGCCCTGCCTCGGT |
|  | pCMV-Myc-HBc T162A | F | CTAGAAGAAGAGCTCCCTCGCCTCG |
|  |  | R | CGAGGCGAGGGAGCTCTCTTCTAG |
|  | pCMV-Myc-HBc S164A | F | GAAGAACTCCCGCGCCTCGCAGACG |
|  |  | R | CGTCTGCGAGGCGCGGGAGTTCTTC |
|  | pCMV-Myc-HBc S172A | F | GCAGATCTCAAGCGCCGCGTCGCAG |
|  |  | R | CTGCGACGCGGCGCTTGAGATCTGC |
|  | pCMV-Myc-HBc Y132A | F | CTCCTCCAGCCGCTAGACCACCAAA |
|  |  | R | TTTGGTGGTCTAGCGGCTGGAGGAG |
|  | pCMV-Myc-HBc SST-SAAAAA | F | GACCGAGGCAGGTCCCCTAGAAGAAGAG |
|  |  | R | CTCTTCTTCTAGGGGACCTGCCTCGGTC |
|  | pCMV-Myc-HBc SST-AASAAA | F | AGAAGAGCTCCCTCGCCTCGCAGACGCA |
|  |  | R | TGCGTCTGCGAGGCGAGGGAGCTCTTCT |
|  | pCMV-Myc-HBc SST-AAAASA | F | CGCAGAGCTCAATCGCCGCGTCGCAGAA |
|  |  | R | TTCTGCGACGCGGCGATTGAGCTCTGCG |
| shRNA | shcontrol |  | GTTTCCGAACGTGTCACGTCTTCCTGTCAGAACGTGACA  CGTTCGGAGAACTTTTT |
|  | shPin1 #1 |  | CCGGCCACCGTCACACAGTATTTATCTCGAGATAAATACT  GTGTGACGGTGGTTTTT |
|  | shPin1 #2 |  | CCGGGCCATTTGAAGACGCCTCGTTCTCGAGAACGAGGC  GTCTTCAAATGGCTTTTT |
|  | shPin1 #3 |  | CCGGCCAGAAGATCAAGTCGGGAGACTCGAGTCTCCCGA  CTTGATCTTCTGGTTTTT |
|  | shPin1 #4 |  | CCGGAGGAGAAGATCACCCGGACCACTCGAGTGGTCCGG  GTGATCTTCTCCTTTTTT |
|  | shPin4 #1 |  | CCGGGTCAGACACATTCTATGTGAACTCGAGTTCACATA  GAATGTGTCTGACTTTTTG |

^a^ Lower case letters in the primer sequences denote inserted restriction endonuclease sites.

^b^ Inserted *Hin*d III site, ^c^ Inserted *Bam*H I site, ^d^ Inserted *Msc* I site, ^e^ Inserted *Kpn* I site.
